# ATP13A3 is a major component of the enigmatic mammalian polyamine transport system

**DOI:** 10.1074/jbc.RA120.013908

**Published:** 2020-12-17

**Authors:** Norin Nabil Hamouda, Chris Van den Haute, Roeland Vanhoutte, Ragna Sannerud, Mujahid Azfar, Rupert Mayer, Álvaro Cortés Calabuig, Johannes V. Swinnen, Patrizia Agostinis, Veerle Baekelandt, Wim Annaert, Francis Impens, Steven H.L. Verhelst, Jan Eggermont, Shaun Martin, Peter Vangheluwe

**Affiliations:** 1 Laboratory of Cellular Transport Systems, Department of Cellular and Molecular Medicine, KU Leuven, Leuven, Belgium; 2 Laboratory for Neurobiology and Gene Therapy, Department of Neurosciences, KU Leuven, Leuven, Belgium; 3 Leuven Viral Vector Core, KU Leuven, Leuven, Belgium; 4 Laboratory of Chemical Biology, Department of Cellular and Molecular Medicine, KU Leuven, Leuven, Belgium; 5 VIB-KU Leuven Laboratory of Membrane Trafficking, Department of Neurosciences, KU Leuven, Leuven, Belgium; 6 Department for Biomolecular Medicine, VIB Center for Medical Biotechnology, VIB Proteomics Core, Ghent University, Ghent, Belgium; 7 Genomics Core Leuven, KU Leuven, Leuven, Belgium; 8 Laboratory of Lipid Metabolism and Cancer, Department of Oncology, LKI – Leuven Cancer Institute, KU Leuven, Leuven, Belgium; 9 Laboratory of Cell Death Research & Therapy, Department of Cellular and Molecular Medicine, KU Leuven, Leuven, Belgium; 10 Department of Oncology, VIB-KU Leuven Center for Cancer Biology, KU Leuven, Leuven, Belgium; 11 Chemical Proteomics, Leibniz Institute for Analytical Sciences ISAS, Dortmund, Germany

**Keywords:** ATP13A3, P-type ATPase, transporter, P5B-ATPase, polyamine, polyamine transport system, putrescine, APols, amphipol, BODIPY, boron dipyrromethene, BSA, bovine serum albumin, BV, benzyl viologen, CHO, Chinese hamster ovary, DAPI, 4',6-diamidino-2-phenylindole, DCM, dichloromethane, DFMO, difluoromethylornithine, DPBS, Dulbecco's PBS, DPBS-T, DPBS containing 0.1% Tween 20, EEA1, early endosomal antigen 1, ESI-MS, electrospray ionization-mass spectrometry, FA, formic acid, FBS, fetal bovine serum, FDR, false discovery rate, LAMP1, lysosomal-associated membrane protein 1, MGBG, methylglyoxal bis-(guanylhydrazone), PTS, polyamine transport system, PUT, putrescine, RAB7/11, ras-associated binding protein 7/11, SPD, spermidine, SPM, spermine

## Abstract

Polyamines, such as putrescine, spermidine, and spermine, are physiologically important polycations, but the transporters responsible for their uptake in mammalian cells remain poorly characterized. Here, we reveal a new component of the mammalian polyamine transport system using CHO-MG cells, a widely used model to study alternative polyamine uptake routes and characterize polyamine transport inhibitors for therapy. CHO-MG cells present polyamine uptake deficiency and resistance to a toxic polyamine biosynthesis inhibitor methylglyoxal bis-(guanylhydrazone) (MGBG), but the molecular defects responsible for these cellular characteristics remain unknown. By genome sequencing of CHO-MG cells, we identified mutations in an unexplored gene, *ATP13A3*, and found disturbed mRNA and protein expression. *ATP13A3* encodes for an orphan P5B-ATPase (ATP13A3), a P-type transport ATPase that represents a candidate polyamine transporter. Interestingly, ATP13A3 complemented the putrescine transport deficiency and MGBG resistance of CHO-MG cells, whereas its knockdown in WT cells induced a CHO-MG phenotype demonstrated as a decrease in putrescine uptake and MGBG sensitivity. Taken together, our findings identify ATP13A3, which has been previously genetically linked with pulmonary arterial hypertension, as a major component of the mammalian polyamine transport system that confers sensitivity to MGBG.

The polyamines, putrescine (PUT), spermidine (SPD), and spermine (SPM), are abundant aliphatic polycations that are required for various cell functions including proliferation, differentiation, apoptosis, protein post-translational modifications, and ion channel regulation (1, 2). Polyamines provide cardioprotection and extend life span in several model organisms (3, 4), whereas excessive concentrations of polyamines induce cellular toxicity, possibly through the formation of toxic metabolites (5). Therefore, the cellular polyamine homeostasis is tightly controlled by fine-tuning polyamine biosynthesis, degradation, export, and cellular uptake of exogenous polyamines *via* the polyamine transport system (PTS) (2).

Polyamine synthesis starts from ornithine that is converted to PUT by ornithine decarboxylase, followed by PUT metabolism to SPD and SPM *via* SPD and SPM synthase, respectively (Fig. S1) (2). This pathway is strictly regulated mainly through controlling the levels and activity of the rate-limiting enzyme ornithine decarboxylase *via* antizyme and antizyme inhibitor (Fig. S1) (6). Polyamine synthesis can also be prevented by synthetic blockers such as difluoromethylornithine (DFMO), a selective inhibitor of ornithine decarboxylase, or methylglyoxal bis-(guanylhydrazone) (MGBG), an SPD analog that inhibits the formation of decarboxylated S-adenosylmethionine, a precursor of SPD and SPM (Fig. S1) (7). Inhibition of polyamine synthesis by DFMO leads to an increased cellular polyamine uptake (8, 9, 10) and increased ornithine decarboxylase and S-adenosylmethionine decarboxylase synthesis (8), indicating that polyamine production and uptake exert complementary functions.

So far, the mechanism of cellular polyamine uptake and the identity of the mammalian PTS remain largely unknown (6, 9, 11) although polyamine transporters represent interesting cancer targets (12). One of the best-studied models used to characterize the mammalian PTS includes a mutant Chinese hamster ovary (CHO) cell line that was generated by random mutagenesis followed by selection for MGBG resistance (hence named CHO-MG) (13). These cells exhibit a distinct phenotype manifested by an impaired polyamine uptake and a better survival against MGBG toxicity due to a reduced cellular uptake of MGBG (14). The cell model has been extensively used to study pathways of the enigmatic mammalian PTS (13, 14, 15, 16, 17, 18) and to test polyamine transport inhibitors for therapy (19, 20, 21, 22). However, despite serious efforts, the defective polyamine transporter(s) in the CHO-MG model remain(s) to be identified.

Based on studies in CHO-MG cells and other models, several polyamine transport routes have been proposed to account for experimental observations of cellular polyamine uptake, but a unifying theory is lacking, presumably because of the existence of multiple parallel systems (12). Potential plasma membrane polyamine transporters include the solute carrier transporter, SLC3A2, with PUT selectivity (23, 24). An alternative pathway involves the endocytic internalization of extracellular polyamines *via* heparan sulfate groups of plasma membrane proteins called glypicans (25, 26). Also, a vesicular SLC18B1 importer has been reported presenting SPD and SPM selectivity (27). Recently, we characterized the ubiquitous P5B-ATPase, ATP13A2, as a polyamine transporter in the late endosomal/lysosomal compartment that preferentially sequesters SPM and SPD out of the late endosomal/lysosomal lumen into the cytosol (28). ATP13A2 removes polyamines from the lysosome, which benefits lysosomal health and functionality. This process is compatible with the glypican-dependent endosomal uptake route that contributes to the cellular uptake of polyamines complementing the polyamine synthesis in the cytosol. ATP13A2 may mediate cellular polyamine uptake *via* a two-step mechanism involving cellular entry of polyamines through endocytosis, followed by sequestration of polyamines out of the late endosomal/lysosomes by ATP13A2 (28). It remains unknown whether the other orphan P5B-ATPases, ATP13A3-5, may also be polyamine transporters of the mammalian PTS (29).

We, therefore, hypothesized that the underlying molecular defect of the CHO-MG phenotype might be due to a dysfunction of one or more members of the P5B-ATPases. In CHO-MG cells, we identified mutations in the coding sequence of the *ATP13A3* gene, which encodes for a P5B-ATPase expressed in the early and recycling endosomes (29). We demonstrated ATP13A3 expression deficiency at both the mRNA and protein levels. Importantly, reintroducing WT ATP13A3 restores the polyamine uptake and MGBG resistance phenotype of CHO-MG cells, whereas ATP13A3 knockdown in WT cells induces these phenotypes. Therefore, ATP13A3 represents a novel member of the mammalian PTS.

## Results

### CHO-MG cells exhibit MGBG resistance and impaired BODIPY–PUT uptake

**Figure 1:**
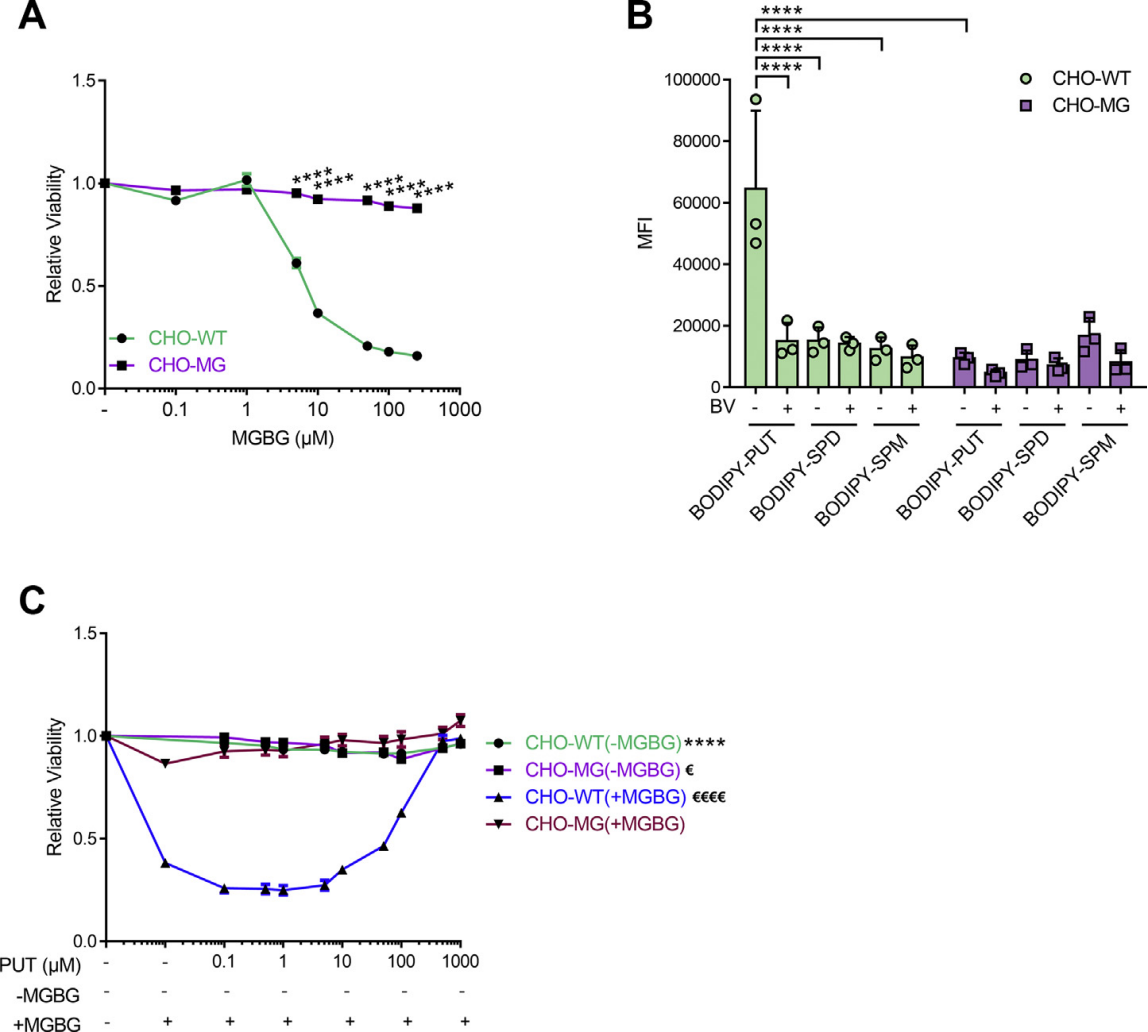
**CHO-MG cells exhibit MGBG resistance and impaired BODIPY–PUT uptake.***A* and *C*, cells were treated for 24 h with the indicated concentrations of MGBG alone (*A*), or PUT with or without 50-μM MGBG (*C*). CellTiter 96 AQ_ueous_ One Solution Cell Proliferation Assay (MTS) was used to assess cell viability and dose–response curves were plotted (n = 3). *B*, cells were treated with 5-μM BODIPY–polyamines for 90 min at 37 °C with or without 90 min of 1-mM BV pretreatment to inhibit polyamine uptake. Uptake was measured in terms of the mean fluorescence intensity (MFI) up to 1 × 10^4^ events (debris free) per condition on the flow cytometer (n = 3). Data represent the mean ± SEM (*A* and *C*) or mean ± SD (*B*), and individual data points (representing replicates) are overlaid on bar graph plots (^*€*^*p* < 0.05, ^∗∗∗∗^^/€€€€^*p* < 0.0001, ^∗∗∗∗^*versus* CHO-WT(+MGBG), ^€€€€^*versus* CHO-MG(+MGBG)). Analyses were performed using two-way ANOVA and Bonferroni *post hoc* corrections. BODIPY, boron dipyrromethene; BV, benzyl viologen; MGBG, methylglyoxal bis-(guanyl hydrazone); SPD, spermidine; SPM, spermine; PUT, putrescine.

First, we confirmed *via* viability assays the resistance of CHO-MG cells against MGBG-induced toxicity as compared with CHO control cells (CHO-WT) (Fig. 1*A*) (9, 13, 14). Furthermore, we verified whether the PTS was defective in the CHO-MG cell line. We assessed the cellular polyamine uptake capacity using boron dipyrromethene (BODIPY)-conjugated SPD and SPM (Fig. 1*B*) that are taken up by the PTS (30) and transported by ATP13A2 (28), but both CHO cell lines exhibited similar levels of BODIPY–SPD and BODIPY–SPM uptake (Fig. 1*B*). Also, CHO-WT and CHO-MG models were equally sensitive to the toxicity of high SPD and SPM concentrations (Fig. S2). Because no obvious SPD or SPM phenotype was observed in CHO-MG, we next evaluated PUT uptake and toxicity. We, therefore, synthesized BODIPY-labeled PUT for this study and observed that its uptake was approximately 5-fold lower in CHO-MG than in CHO-WT (Fig. 1*B*), confirming a defective PTS in CHO-MG. Benzyl viologen (BV), a known inhibitor of the PTS (31), blocked BODIPY–PUT uptake in CHO-WT to the levels observed in CHO-MG cells (Fig. 1*B*). We then investigated the effect of increasing concentrations of exogenous PUT on the toxicity of 50-μM MGBG, a concentration that caused a significant reduction in CHO-WT viability, as previously shown in Figure 1*A*. Supplementation of PUT on itself did not induce toxicity in either of the two CHO models (Fig. 1*C*), in line with previous observations (28), whereas PUT reduced MGBG toxicity in CHO-WT in a dose-dependent manner reaching complete prevention at 1-mM PUT (Fig. 1*C*). Because MGBG is taken up by the PTS (7, 13, 14), our results point to a competition between PUT and MGBG uptake *via* the same transport system.

Overall, CHO-MG cells are resistant to MGBG and present a reduced BODIPY–PUT uptake capacity, which may relate to a defective polyamine transporter of MGBG and PUT.

### ATP13A3 deficiency in CHO-MG cells

**Figure 2:**
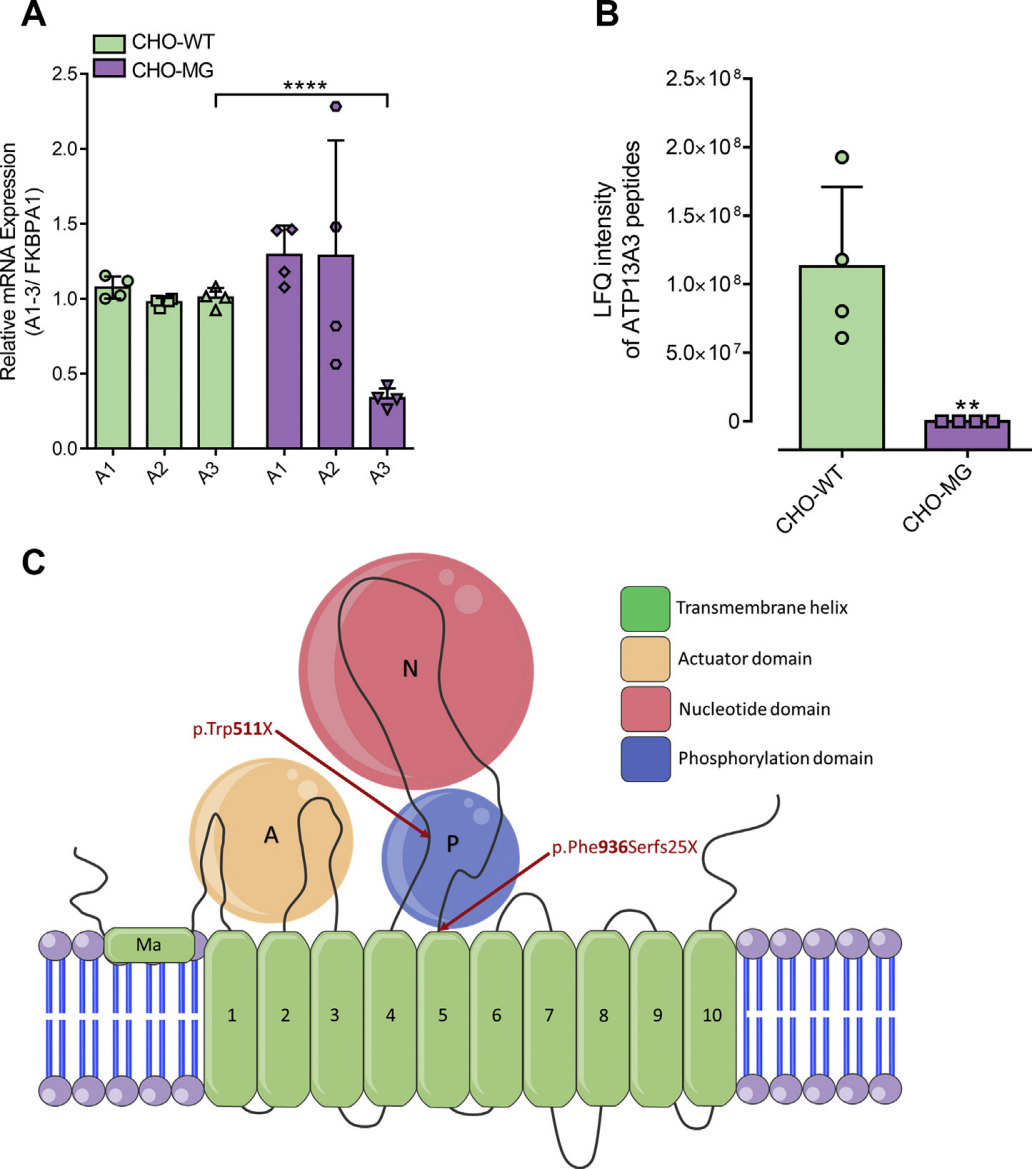
**ATP13A3 is downregulated in CHO-MG cells.***A*, ATP13A1-3 mRNA levels were measured with quantitative RT-PCR using SYBR Green master mix (n = 4). *B*, protein expression of ATP13A3 was checked *via* proteomic analysis (n = 4). *C*, ATP13A3 topology model with the identified mutations in CHO-MG cells. Data represent the mean ± SD (*A* and *B*) and individual data points (representing replicates) are overlaid on bar graph plots (^∗∗^*p* < 0.01, ^∗∗∗∗^*p* < 0.0001). Analyses were performed using one-way ANOVA and Bonferroni *post hoc* corrections (*A*) and unpaired t-test (*B*). A1-A3, ATP13A1-3; FKBPA1, FKBP prolyl isomerase 1A.

Based on the recent discovery that ATP13A2 is a polyamine transporter that regulates the cellular polyamine content (28), we hypothesized that ATP13A2, or one of the other related P5-type orphan transporters such as ATP13A1 (a P5A-ATPase) or ATP13A3-5 (P5B-ATPases) (29), may be implicated in the CHO-MG phenotype. The Chinese hamster (*Cricetulus griseus*) genome does not contain an *ATP13A5* gene (32), so we compared the relative mRNA expression levels of the P5A-ATPase, ATP13A1, and the P5B-ATPases, ATP13A2-4, between CHO-WT and CHO-MG *via* quantitative RT-PCR (Fig. 2*A*), for which the primer sets were validated (Fig. S3A). Whereas the relative mRNA levels of ATP13A1 and ATP13A2 were not significantly different, ATP13A3 mRNA was significantly decreased in CHO-MG compared with CHO-WT cells (Fig. 2*A*). Importantly, we confirmed the decreased expression of ATP13A3 in CHO-MG cells at the protein level *via* proteomic analysis (Fig. 2*B* and Fig. S3*B*). ATP13A4 mRNA expression was hardly detected in the CHO models although the ATP13A4 primers were validated with cDNA derived from hamster brain, the tissue that shows the highest ATP13A4 expression in mice (33) (Fig. S3*C*). The low ATP13A4 expression in CHO cells is in line with the poor ATP13A4 expression in human ovaries (34).

As CHO-MG cells were generated by random mutagenesis (13), we analyzed the DNA sequence of the CHO-MG *ATP13A3* gene. Genome sequencing revealed 54 DNA mutations in the CHO-MG *ATP13A3* gene as compared with the reference CHO genome (RefSeq Assembly GCF_000223135.1). Only two of the 54 mutations were found in the coding sequence and both of them were heterozygous. The first coding sequence mutation is located on nucleotide 45284 of contig NW_003615339.1 (genotype = 0/1; read depth for each allele = 21,33; read depth = 54; genotype quality = 99; phred-scaled genotype likelihoods rounded to the closest integer = 1216,0,715), leading to a nonsense mutation at the protein level converting the Trp codon 511 (TGG) to a stop codon (TGA) (p.Trp511X) (Fig. 2*C*). The second coding sequence mutation results in a frameshift at the protein level at position 936 (p.Phe936Serfs25X) (Fig. 2*C*). Here, nucleotide 57302 in contig NW_003615339.1 is deleted (genotype = 0/1; read depth for each allele = 25,27; read depth = 52; genotype quality = 99; phred-scaled genotype likelihoods rounded to the closest integer = 855,0,777), removing the first thymine in the Phe codon (TTC) at position 936 of the ATP13A3 protein sequence. Both mutations in the coding sequence of *ATP13A3* are compatible with the decreased mRNA and protein expression of ATP13A3 in CHO-MG cells.

### Impaired ATP13A3 transport activity underlies the CHO-MG phenotype

**Figure 3:**
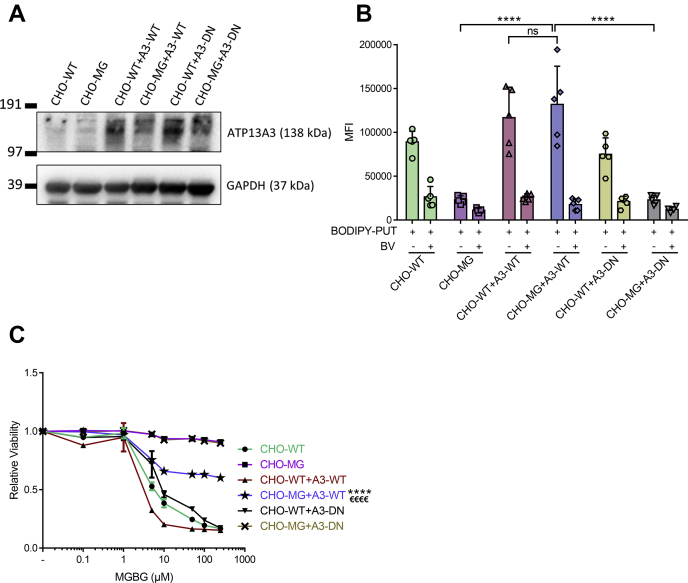
**Expression of WT ATP13A3 restores the CHO-MG phenotype.***A*, stable cell lines were generated by lentiviral transduction to overexpress WT (A3-WT) or a catalytically dead mutant (A3-DN) of ATP13A3. Expression of the viral vectors was verified by immunoblotting using an ATP13A3 selective antibody, while the loading was monitored by a selective antibody of the house-keeping protein, GAPDH. *B*, cells were treated with 5-μM BODIPY–polyamines for 90 min at 37 °C with or without 90 min of 1-mM BV before treatment to inhibit polyamine uptake. Uptake was measured in terms of the mean fluorescence intensity (MFI) up to 1 × 10^4^ events (debris free) per condition on the flow cytometer (n = 5–9). *C*, cells were treated for 24 h with different doses of MGBG. Cell viability was assessed using CellTiter 96 AQ_ueous_ One Solution Cell Proliferation Assay (MTS), and dose–response curves were plotted (n = 3). Data represent the mean ± SD (*B*) and individual data points (representing replicates) are overlaid on bar graph plots, or the mean ± SEM (*C*) (^∗^^∗^^∗^^∗^^/€€€€^*p* < 0.0001, ns = not significant, ^∗^^∗^^∗^^∗^*versus* CHO-WT + A3-WT, ^€€€€^*versus* CHO-MG and CHO-MG + A3-DN). Analyses were performed using two-way ANOVA and Bonferroni *post hoc* corrections. A3-DN, overexpression of ATP13A3 catalytically dead mutant D498N; A3-WT, overexpression of WT ATP13A3; BODIPY, boron dipyrromethene; BV, benzyl viologen; MGBG, methylglyoxal bis-(guanyl hydrazone); PUT, putrescine.

Next, we verified whether the impaired ATP13A3 expression is responsible for the CHO-MG phenotype by restoring ATP13A3 levels *via* lentiviral transduction. We generated stable CHO-WT and CHO-MG cells expressing comparable levels of human WT (A3-WT) or a catalytically dead mutant of ATP13A3 that is defective in the autophosphorylation activity (D498N; A3-DN) and hence predicted to be transport deficient (Fig. 3*A*) (29). Note that the ATP13A3 antibody fails to detect a clear band in CHO-WT, indicating that the antibody may react weaker with the endogenous hamster ATP13A3 protein than with the overexpressed human ATP13A3 protein (9 residues are different in the epitope region), and/or the endogenous signal may be overshadowed by the relatively higher overexpression in CHO-WT+A3-WT. Importantly, compared with the nontransduced CHO-MG cells, there was a significant increase in the uptake of BODIPY–PUT in CHO-MG+A3-WT cells, comparable with the uptake level observed in CHO-WT+A3-WT cells (Fig. 3*B*), demonstrating successful recovery of BODIPY–PUT uptake. However, this rescue was not observed in CHO-MG+A3-DN cells expressing the catalytically dead ATP13A3 mutant (Fig. 3*B*), indicating that the transport function of ATP13A3 was essential for the recovery of BODIPY–PUT uptake. Note that the uptake of BODIPY–PUT in CHO-WT+A3-WT cells, exhibiting the highest ATP13A3 expression, still falls within the linear range (Fig. S4). The resistance to MGBG-induced toxicity was also significantly reduced by 40% in CHO-MG+A3-WT cells as compared with the nontransduced CHO-MG and CHO-MG+A3-DN cell lines (Fig. 3*C*).

**Figure 4:**
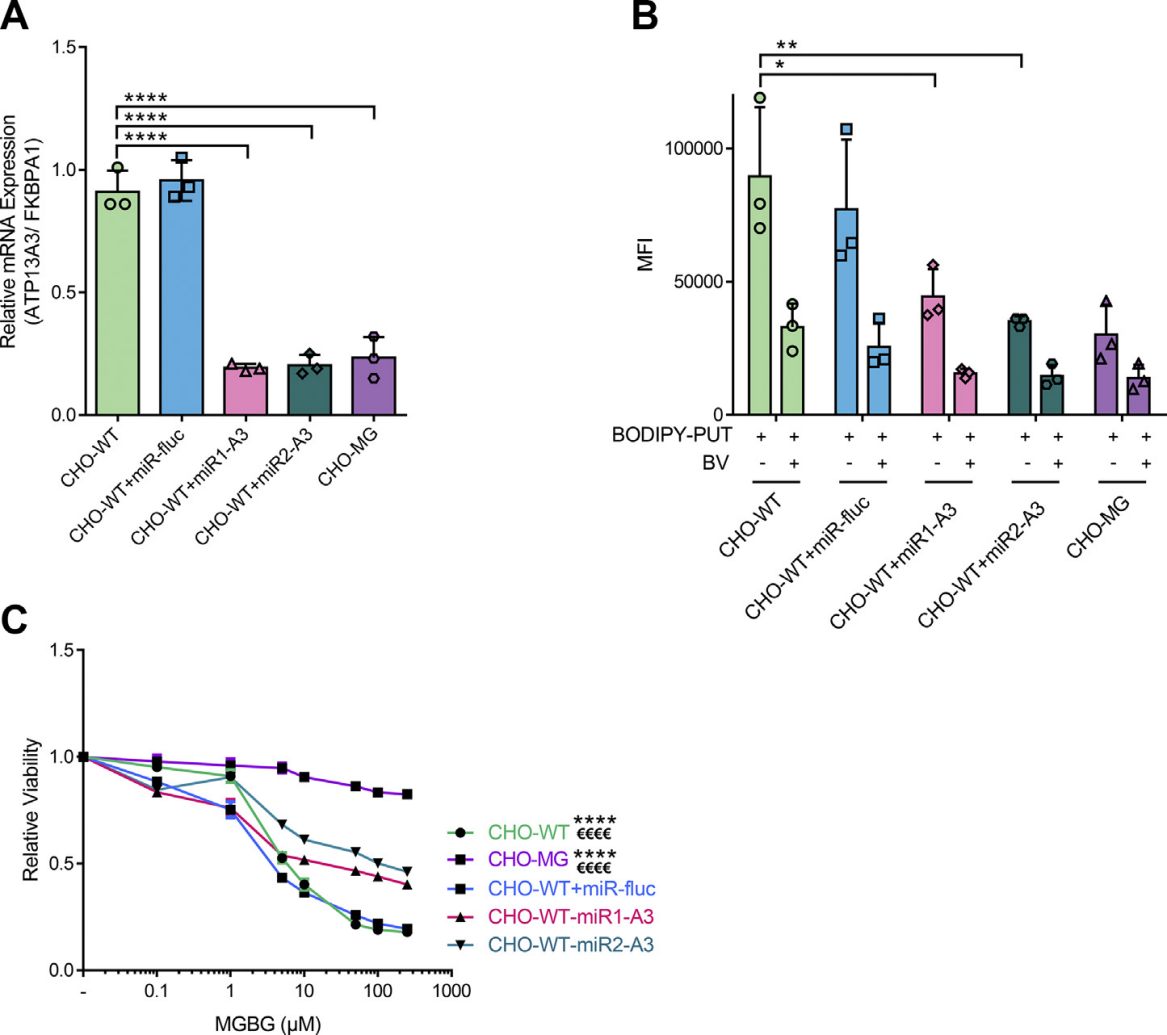
**Depletion of ATP13A3 in CHO-WT cells causes a CHO-MG phenotype.***A*, stable cell lines were generated by lentiviral transduction to knock down ATP13A3 in CHO-WT cells. Efficiency of the knockdown was verified at the mRNA level with quantitative PCR (n = 3). *B*, cells were treated with 5-μM BODIPY–polyamines for 90 min at 37 °C with or without 90 min of 1-mM BV before treatment to inhibit polyamine uptake. Uptake was measured in terms of the mean fluorescence intensity (MFI) up to 1 × 10^4^ events (debris free) per condition on the flow cytometer (n = 3). *C*, cells were treated for 24 h with different doses of MGBG. Cell viability was assessed using CellTiter 96 AQ_ueous_ One Solution Cell Proliferation Assay (MTS), and dose–response curves were plotted (n = 3). Data represent the mean ± SD (*A* and *B*) and individual data points (representing replicates) are overlaid on bar graph plots, or the mean ± SEM (*C*) (^∗^*p* < 0.05, ^∗^^∗^*p* < 0.01, ^∗∗∗∗^^/€€€€^*p* < 0.0001, ^∗∗∗∗^*versus* CHO-WT + A3-KD1, ^€€€€^*versus* CHO-WT + A3-KD2). Analyses were performed using two-way ANOVA and Bonferroni *post hoc* corrections. A3, ATP13A3; A3-KD, knockdown of ATP13A3; BODIPY, boron dipyrromethene; BV, benzyl viologen; FKBPA1, FKBP prolyl isomerase 1A; MGBG, methylglyoxal bis-(guanyl hydrazone); miR-fluc, expression of microRNA against firefly luciferase; PUT, putrescine.

To further establish ATP13A3 dysfunction as a key contributor in the CHO-MG phenotype, we induced stable shRNA-mediated knockdown of ATP13A3 in the CHO-WT background and checked if that would lead to a phenotype comparable with CHO-MG cells. *Via* quantitative RT-PCR, we observed a major reduction in ATP13A3 expression in two independent knockdown models (Fig. 4*A*), which reduced the uptake of BODIPY–PUT to the same level as in CHO-MG cells (Fig. 4*B*) and significantly increased the resistance of the cells against MGBG by 40% (Fig. 4*C*), in line with the rescue results (Fig. 3*C*).

Together, the complementary data in the rescue and knockdown cell models convincingly demonstrate that defective ATP13A3-mediated transport underlies the impaired BODIPY–PUT uptake in CHO-MG cells and is at least partially responsible for the MGBG resistance phenotype.

### ATP13A3-dependent transport presents broad polyamine specificity

**Figure 5:**
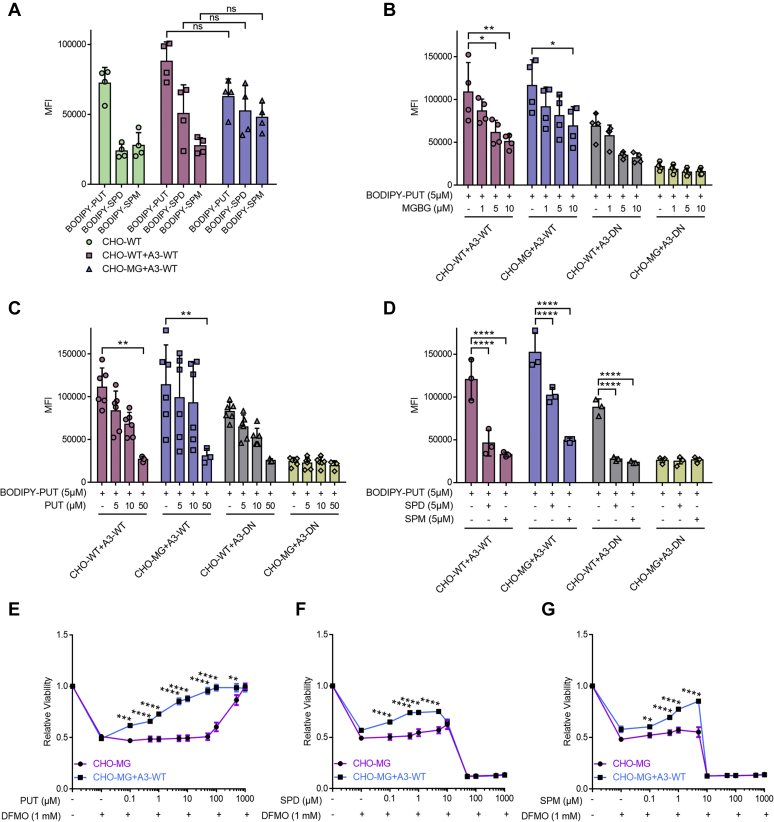
**ATP13A3-dependent transport exhibits a broad specificity toward PUT, SPD, and SPM.***A*–*D*, cells were treated with only 5-μM BODIPY–PUT, BODIPY–SPD, or BODIPY–SPM (n = 4) (*A*), or with 5-μM BODIPY–PUT combined with different concentrations (1 μM, 5 μM, 10 μM, or 50 μM) of MGBG (n = 4) (*B*), nonfluorescent PUT (n = 3–6) (*C*) or nonfluorescent SPD/SPM (n = 3) (*D*) for 90 min at 37 °C. Uptake was measured in terms of the mean fluorescence intensity (MFI) up to 1 × 10^4^ events (debris free) per condition on the flow cytometer. *E*–*G*, cells were treated for 72 h with the indicated concentrations of PUT (*E*), SPD (*F*), and SPM (*G*) combined with 1-mM DFMO. CellTiter 96 AQ_ueous_ One Solution Cell Proliferation Assay (MTS) was used to assess cell viability, and dose–response curves were plotted (n = 3). Data represent the mean ± SD (*A*–*D*), and individual data points (representing replicates) are overlaid on bar graph plots, or the mean ± SEM (*E*-*G*) (^∗^*p* < 0.05, ^∗^^∗^*p* < 0.01, ^∗^^∗^^∗^*p* < 0.001, ^∗^^∗^^∗^^∗^*p* < 0.0001, ns = not significant). Analyses were performed using two-way ANOVA and Bonferroni *post hoc* corrections. A3-DN, overexpression of ATP13A3 catalytically dead mutant D498N; A3-WT, overexpression of WT ATP13A3; BODIPY, boron dipyrromethene; BV, benzyl viologen; DFMO, difluoromethylornithine; MGBG, methylglyoxal bis-(guanyl hydrazone); PUT, putrescine; SPD, spermidine; SPM, spermine.

We examined the likely substrate specificity of ATP13A3-dependent transport *via* cellular uptake experiments. Overexpression of WT ATP13A3 in CHO-MG caused no significant difference in the uptake of BODIPY–PUT, BODIPY–SPD, and BODIPY–SPM as compared with CHO-WT+A3-WT (Fig. 5*A*). *Via* competition assays, we observed that the unlabeled PUT competed with BODIPY–PUT uptake in cells expressing WT ATP13A3 (Fig. 5*C*), further confirming that PUT is most likely a substrate transported through ATP13A3. Interestingly, nonfluorescent SPD and SPM also competed with the uptake of BODIPY–PUT in all cell lines with functional ATP13A3, possibly hinting at a broad polyamine substrate specificity (Fig. 5*D*). Moreover, MGBG showed competition against BODIPY–PUT uptake in a dose-dependent manner in CHO-WT+A3-WT and CHO-MG+A3-WT (Fig. 5*B*), confirming that MGBG and BODIPY–PUT follow the same uptake route *via* ATP13A3. Also, SPD and SPM protected against MGBG toxicity in CHO-WT cells (Fig. S5, *A*–*B*).

Next, we independently verified substrate specificity in viability experiments with DFMO, an inhibitor of ornithine decarboxylase, the rate-limiting step in polyamine synthesis. Blocking endogenous polyamine synthesis by DFMO (Fig. S1) makes cells more dependent on cellular uptake of polyamines (9). Interestingly, ATP13A3 expression significantly reduced DFMO toxicity in the CHO-MG background, mainly when exogenous PUT was provided, and more mildly when SPD or SPM was administered (Fig. 5, *E*–*G*), demonstrating that polyamine uptake *via* ATP13A3 complements a lower ornithine decarboxylase activity. Note that PUT exhibits no toxicity in the concentration range we used, whereas SPD and SPM become toxic at higher concentrations, which explains why ATP13A3 protection against DFMO fades at higher concentrations of SPD and SPM (Fig. 5, *E*–*G*). Overall, our data indicate that ATP13A3-dependent transport presents a broad specificity to polyamines including PUT, SPD, and SPM, as well as MGBG.

### Cellular uptake of BODIPY–PUT involves endocytosis

**Figure 6:**
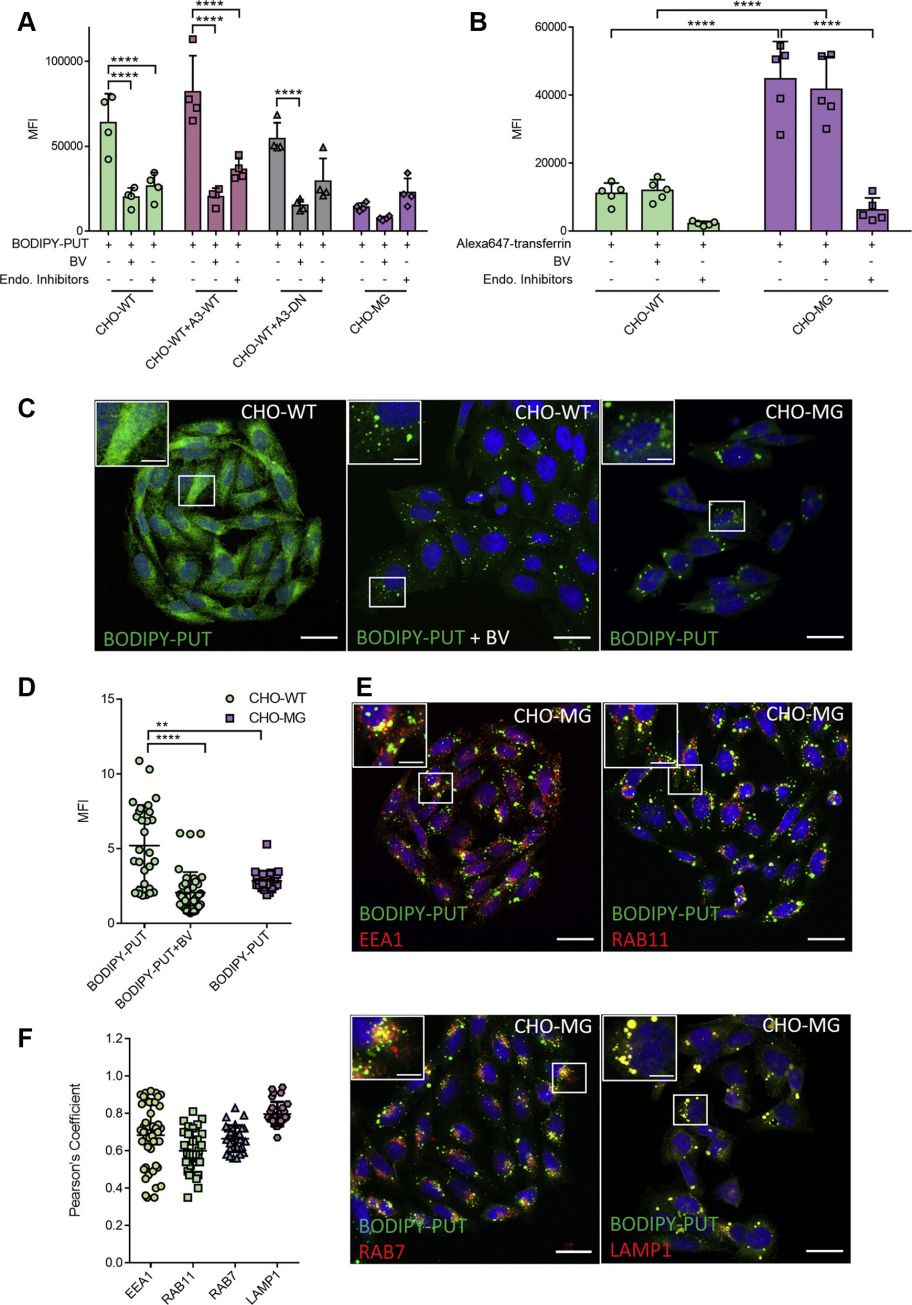
**BODIPY–PUT is taken up *via* endocytosis.***A*, cells were treated with 5-μM BODIPY–PUT for 90 min at 37 °C with or without 30 min pretreatment of 1-mM BV or endocytosis inhibitors (100-μM Dynasore, 50-μM genistein, and 50-μM Pitstop-2). Uptake was measured in terms of the mean fluorescence intensity (MFI) up to 1 × 10^4^ events (debris free) per condition on the flow cytometer (n = 3–4). *B*, cells were treated with BV or endocytosis inhibitors, while being starved for 30 min. Afterward, they were incubated at 4 °C for 15 min, treated with 50 μg/ml Alexa647-transferrin for 20 min, and incubated at 37 °C, 5% CO_2_ for 15 min. Uptake was measured in terms of the mean fluorescence intensity (MFI) up to 1 × 10^4^ events (debris-free) per condition on the flow cytometer (n = 5). *C* and *E*, cells were treated with BODIPY–PUT for 90 min at 37 °C with or without 30 min pretreatment of 1-mM BV and incubated with primary antibodies for EEA1, RAB11, RAB7, and LAMP1 (1:200) followed by staining with Alexa Fluor 594 goat anti-rabbit antibody (1:1000) for 60 min and the nuclear stain DAPI (200 ng/ml) for 15 min (n=3–5). Confocal microscopy images (scale bar = 20 μm; *boxed* areas are enlarged in the inset with scale bar = 5 μm) of BODIPY–PUT in CHO-WT ± BV and CHO-MG (*C*), and BODIPY–PUT colocalization with endosomal markers in CHO-MG (*E*) are shown. *D*, BODIPY–PUT, represented in panel *C*, was quantified by measuring the mean fluorescence intensities (MFI) of BODIPY normalized to that of DAPI (CHO-WT + BODIPY–PUT = 29 images; CHO-WT BODIPY–PUT + BV = 39 images; CHO-MG BODIPY–PUT = 25 images). *F*, colocalization of BODIPY–PUT with endosomal markers, demonstrated in panel *E*, was analyzed in terms of Pearson’s coefficient of BODIPY–PUT with EEA1 (49 images), RAB11 (30 images), RAB7 (29 images), and LAMP1 (30 images). Data represent the mean ± SD, and individual data points (representing replicates) are overlaid on bar graph plots (^∗∗^*p* < 0.01, ^∗∗∗^^∗^*p* < 0.0001), generated with two-way ANOVA and Bonferroni *post hoc* corrections. A3-DN, overexpression of ATP13A3 catalytically dead mutant D498N; A3-WT, overexpression of WT ATP13A3; BODIPY, boron dipyrromethene; BV, benzyl viologen; EEA1, early endosomal antigen 1; LAMP1, lysosomal-associated membrane protein 1; PUT, putrescine; RAB7/11, ras-associated binding protein 7/11.

Because ATP13A3 localizes to the early and recycling endosomes (29), we investigated whether endocytosis is involved in the uptake of BODIPY–PUT by testing the effect of endocytosis inhibitors. Administration of a cocktail of Dynasore, genistein, and Pitstop 2, which collectively inhibits the clathrin-mediated, clathrin-independent, and caveolin-mediated endocytic pathways (28), significantly reduced BODIPY–PUT uptake in CHO-WT and CHO-WT+A3-WT but not in CHO-MG cells (Fig. 6*A*), demonstrating the contribution of endocytosis in the uptake process. Also, we observed *via* confocal microscopy that the fluorescence intensity of BODIPY–PUT was significantly higher and more widespread in CHO-WT than in CHO-MG cells where the signal was confined to vesicular structures (Fig. 6, *C*–*D*) that colocalized with various endosomal markers (Fig. 6, *E*–*F*). These observations in CHO-MG cells are in line with the impaired transport of endocytosed BODIPY–PUT to the cytosol as a consequence of ATP13A3 dysfunction. We also checked whether the lower BODIPY–PUT uptake in CHO-MG cells is not merely the result of impaired endocytosis by measuring uptake of Alexa647-transferrin (35) that is sensitive to the same endocytosis inhibitor cocktail (Fig. 6*B*). Remarkably, we observed that the endocytosis of Alexa647-transferrin in CHO-MG cells was 5-fold higher than in CHO-WT (Fig. 6*B*), which is, therefore, not responsible for the lower BODIPY–PUT uptake levels observed in CHO-MG cells.

Although BV is a potent inhibitor of BODIPY–PUT uptake (Fig. 6*A*), it had no effect on Alexa647-transferrin uptake (Fig. 6*B*), indicating that BV may block another stage of the BODIPY–PUT uptake route. Interestingly, BV treatment led to a reduced uptake of BODIPY–PUT that accumulates in vesicular structures, similar to the BODIPY–PUT pattern in ATP13A3-deficient CHO-MG cells (Fig. 6, *C*–*D*). This indicates that BV may inhibit BODIPY–PUT uptake downstream of endocytosis, possibly at the level of ATP13A3.

### ATP13A2 and ATP13A3 fulfill overlapping functions in cellular polyamine uptake

We recently reported that ATP13A2 is a polyamine transporter with a higher affinity for SPM and SPD than PUT, which stimulates the uptake of BODIPY–SPM and BODIPY–SPD in cells, regulates the endogenous polyamine content, and complements DFMO toxicity (28). Also, ATP13A2 deficiency causes increased sensitivity to exogenous SPD or SPM because of lysosomal stress (28). We, therefore, examined whether ATP13A2 may also play a role in the CHO-MG phenotype. However, in CHO-MG cells, the ATP13A2 mRNA levels were not significantly altered (Fig. 2*A*), the uptake of BODIPY–SPD and BODIPY–SPM was not reduced (Fig. 1*B*), and CHO-WT and CHO-MG models presented a similar sensitivity toward SPD or SPM toxicity (Fig. S2, *A*–*B*). Although these data indicate that ATP13A2 deficiency is most likely not involved in the CHO-MG phenotype, we further investigated if ATP13A2 overexpression may partially convert the CHO-MG phenotype to a CHO-WT phenotype. We, therefore, generated stable CHO-WT and CHO-MG cells overexpressing comparable levels of WT ATP13A2 (A2-WT) or a catalytically dead mutant (D508N; A2-DN) (Fig. S6*A*) and observed that not only ATP13A3 (Fig. 3*B*) but also ATP13A2 rescued BODIPY–PUT uptake to comparable levels as CHO-WT (Fig. S6*B*). Yet, ATP13A2 could not recover the resistance to MGBG-induced toxicity (Fig. S6*C*). Moreover, unlike ATP13A3 (Fig. 5*B*), a competition was not observed between MGBG and PUT in CHO-MG cells overexpressing WT ATP13A2 (Fig. S6*D*), suggesting that the human ATP13A2 isoform is not sensitive to MGBG.

## Discussion

In this study, we identified the orphan P5B-ATPase, ATP13A3, as a major factor in the mammalian PTS that is responsible for the polyamine-transport deficiency phenotype of CHO-MG cells, a frequently used model to characterize the mammalian PTS and polyamine transport inhibitors with therapeutic potential (19, 20, 21, 22).

*Via* genome sequencing in CHO-MG cells, we identified two heterozygous mutations in the coding sequence of the *ATP13A3* gene. Both mutations are most likely located *in trans*, affecting the two alleles of the *ATP13A3* gene, because our proteomics analysis indicated that the ATP13A3 protein is lost in CHO-MG as compared with CHO-WT. One DNA mutation causes an early truncation at the protein level that may lead to nonsense-mediated mRNA decay and subsequent loss of ATP13A3 mRNA and protein. Also, a frameshift mutation was identified, which may result in an unstable protein contributing to the complete loss of ATP13A3 protein. *Via* complementary rescue and knockdown experiments, we demonstrated that ATP13A3 represents a critical genetic defect underlying the CHO-MG phenotype of polyamine uptake deficiency. However, ATP13A3 complementation only resulted in a partial but not full reversion of the MGBG phenotype of CHO-MG cells. The partial recovery may indicate that the human ATP13A3 isoform is not completely compatible with the hamster ortholog. Alternatively, CHO-MG cells were generated by random mutagenesis and several rounds of MGBG selection (7, 13), suggesting that also mutations in other genes may contribute to the MGBG resistance phenotype. The surprisingly high endocytosis rate in CHO-MG cells may indeed point to other genetic alterations or compensatory responses that contribute to the phenotype.

CHO-MG cells represent one of the most frequently studied mammalian cell models with a deficient PTS. Because WT ATP13A3, but not a transport-deficient mutant, rescues the polyamine uptake deficiency in CHO-MG cells, we convincingly demonstrate that the orphan P5B-ATPase ATP13A3 is a major component of the mammalian PTS. In the evolution of the vertebrate lineage, ATP13A3 emerged together with ATP13A2 as the result of early gene duplication of an ancestral P5B-ATPase ortholog (29). *Via* an extensive biochemical analysis on purified protein, we recently demonstrated that ATP13A2 is a polyamine transporter that regulates cellular polyamine uptake (28). ATP13A2 shares with ATP13A3 a highly conserved putative substrate-binding site in transmembrane segment M4 that in ATP13A2 impacts on polyamine transport (28, 36), indicating that also ATP13A3 may most likely be a polyamine transporter. Here, we found that ATP13A3 contributes to cellular polyamine uptake *via* a two-step uptake mechanism, first involving endocytosis followed by ATP13A3-mediated transport of endocytosed polyamines to the cytosol. A two-step cellular uptake mechanism has already been described for SPM involving endocytosis *via* heparan sulfate proteoglycans (15) and lysosomal export *via* ATP13A2 (28). The lower fluorescent intensity of BODIPY–PUT in CHO-MG *versus* CHO-WT cells may suggest that a fraction of the endocytosed BODIPY–PUT in ATP13A3-deficient CHO-MG cells is secreted *via* the recycling endosomes, the compartment where ATP13A3 resides (29). Alternatively, the saturation of luminal polyamines in the endosomal system of CHO-MG cells may reduce the level of free plasma membrane–localized polyamine receptors that capture polyamines before endocytosis, such as glypicans, which bind SPM with high affinity (15).

Our data suggest that ATP13A2 and ATP13A3 fulfill overlapping functions in cellular polyamine uptake, but only ATP13A3 mediates MGBG sensitivity. It is also possible that the human and hamster ATP13A2 isoforms present different sensitivities to MGBG. The fact that two ubiquitous P5B transporters, ATP13A2 and ATP13A3, contribute to cellular polyamine uptake may explain why the identification of the mammalian PTS has been challenging despite serious attempts. Also, ATP13A4 and ATP13A5 are closely related isoforms that may represent additional members of the PTS but display a more restricted tissue and cell-type expression (29). Future studies will address the complementarity and redundancy of these various transporters and their interplay with polyamine metabolism, which is under the control of antizyme and antizyme inhibitors (37).

Studying ATP13A3 in combination with other related P5B-ATPases will open the door to better understand the role of polyamine uptake and transport in a broad range of polyamine-dependent cell functions (1, 2) and the regulation of the life span of model organisms, such as mice, *Drosophila melanogaster*, *Caenorhabditis elegans*, and *Saccharomyces cerevisiae* (3, 4). Conversely, blocking the PTS has been proposed for cancer therapy (12, 38), and insights into the role of P5B-ATPases in cellular polyamine uptake will now facilitate a more targeted drug discovery strategy. ATP13A3 expression has been highlighted as a potential biomarker for DFMO-based therapies in pancreatic cancers (39), suggesting that ATP13A3 may be a possible cancer target. Moreover, heterozygous ATP13A3 mutations have been identified in pulmonary arterial hypertension (40, 41, 42). Our study suggests that altered polyamine transport or uptake may play a role in this rare but fatal disorder. Strikingly, ATP13A2 mutations are not implicated in cardiovascular but in neurodegenerative diseases, such as Parkinson’s disease (43, 44), indicating that ATP13A2 and ATP13A3 exert distinct (patho) physiological roles. Indeed, although both isoforms are ubiquitously expressed, slight differences were observed in substrate specificity between ATP13A2 and ATP13A3, and also their intracellular localization is confined to distinct subcompartments of the endocytic pathway (ATP13A2 in late endosomal/lysosomes and ATP13A3 in early and recycling endosomes) (29). Sequence differences in key regulatory domains further indicate that both isoforms may be differentially controlled by distinct signaling pathways (29, 45). Further biochemical experiments on purified protein are needed to conclusively confirm the putative polyamine transport function of ATP13A3 and compare the relative substrate specificity and regulatory mechanisms of ATP13A3 *versus* ATP13A2.

Compelling evidence has shown the involvement of the ATP13A2 transport activity, as well as its yeast P5B ortholog, YPK9, in the homeostasis of heavy metal ions such as Zn^2+^, Mn^2+^, and Fe^3+^ (46, 47, 48, 49). However, biochemical experiments showed that ATP13A2 is a polyamine transporter (28) and that *in vitro* ATP13A2 activity is not regulated by Zn^2+^, Mn^2+^, and Fe^3+^ (28, 45). The effect of P5B ATPases on heavy metal toxicity may, therefore, be an indirect effect that requires the polyamine transport function. Polyamines are heavy metal-chelating agents and exert potent antioxidant effects, which may explain why P5B polyamine transporters provide protection against heavy metal toxicity (50, 51). Further studies are required to examine whether also ATP13A3 exerts a protective effect against heavy metals.

In conclusion, by investigating the molecular defects in a widely used cell model with polyamine transport deficiency, we have identified the orphan P5B-ATPase, ATP13A3, as a novel member of the enigmatic mammalian PTS that contributes to cellular polyamine uptake and that is implicated in disease.

## Experimental procedures

### Materials

We were kindly supplied with MGBG by Prof. Paul van Veldhoven (KU Leuven, Belgium) and BODIPY-conjugated polyamines (SPD and SPM) by Prof. Steven Verhelst (KU Leuven, Belgium) (30).

Treatments purchased from Sigma are BV (#271845), DFMO (#D193), Dynasore (#D7693), Pitstop-2 (#SML1169), PUT (#P7505), SPM (#S3256), and SPD (#S2626). Alexa647-transferrin (#T23366) was obtained from Invitrogen and genistein (#ab120112) from Abcam.

Primary antibodies used were ATP13A2 (#A3361, Sigma), ATP13A3 (#HPA029471, Sigma), early endosomal antigen 1 (EEA1; #E4156, Sigma), GAPDH (#G8795, Sigma), lysosomal-associated membrane protein 1 (LAMP1; #ab24170, Abcam), ras-associated binding protein 7 (RAB7; #ab137029, Abcam), and RAB11 (#71–5300, Thermo Fisher), and secondary antibodies included anti-mouse IgG (#7076S, Cell Signaling Technology) and anti-rabbit IgG (#7074S, Cell Signaling Technology) horseradish peroxidase–linked antibodies, and Alexa Fluor 594 goat anti-rabbit antibody (#A-11037, Thermo Scientific).

All unlabeled and BODIPY-labeled polyamines (PUT, SPD, and SPM) were prepared in 0.1 M Mops-KOH (pH 7.0) to a final stock concentration of 500 mM (200 mM in the case of unlabeled SPM). DFMO was prepared fresh in a cell culture media to a final stock concentration of 500 mM, and MGBG was dissolved in Milli-Q water to a final stock concentration of 100 mM. The endocytosis inhibitors, Dynasore, genistein, and Pitstop-2, were dissolved in dimethyl sulfoxide (#276855, Sigma) to final concentrations of 50 mM, 25 mM, 25 mM, and 10 mg/ml, respectively.

Information about other reagents and kits is mentioned when used throughout this section.

### Cell culture and lentiviral transduction

CHO-MG cells were generously provided by Prof. Wayne Flintoff (University of Western Ontario, Canada), and CHO-WT cells were used as a corresponding control. CHO-WT and CHO-MG cells were cultured, respectively, in Ham’s Nutrient Mixture F12 (supplemented with 5% Eagle's minimum essential medium nonessential amino acid solution; #M7145, Sigma) and RPMI1640 media (supplemented with 2 μg/ml L-proline; #P5607, Sigma) containing 10% fetal bovine serum (FBS; # F7524, Sigma), 10% GlutaMAX (#35050-038, Gibco), and 1% penicillin/streptomycin (#P4458, Sigma) at 37 °C with 5% CO_2_.

Both of the CHO-WT and CHO-MG cell lines were authenticated *via* DNA fingerprinting (Leibniz-Institut DSMZ-Deutsche Sammlung von Mikroorganismen und Zellkulturen GmbH).

Lentiviral transduction of CHO-WT/MG cells was carried out using a lentiviral vector of human ATP13A2 or ATP13A3 to induce overexpression and miRNA-based short hairpin RNA of human ATP13A3 to induce knockdown (52). The protein expression of WT ATP13A2 (A2-WT) or ATP13A3 (A3-WT) and catalytically dead mutants, D508N of ATP13A2 (A2-DN) or D498N of ATP13A3 (A3-DN), in the stable CHO-WT/MG cell lines was verified by immunoblotting, and the knockdown was verified by quantitative RT-PCR.

All cell lines used were checked for *Mycoplasma* contamination using PlasmoTest *Mycoplasma* Detection kit (InvivoGen) and were shown to be negative.

### Cellular viability assay

Cells were cultured in a 96-well plate at a density of 1 × 10^4^ cells per well, except for DFMO experiments for which 5 × 10^3^ cells per well were seeded. The media were discarded the next day and replaced with media containing different concentrations of the treatment of interest. Cell viability was assessed after 24 h, and after 72 h for DFMO treatments using the CellTiter 96 AQ_ueous_ One Solution Cell Proliferation Assay (MTS) (#G3580, Promega) following the manufacturer instructions. Absorbance was assessed at 490 nm using a plate reader (Molecular Devices).

### Cellular polyamine uptake, competition assays, and endocytosis

Cells were cultured in a 12-well plate at a density of 3 × 10^5^ cells per well and were treated the next day for 90 min at 37 °C with 5-μM BODIPY-conjugated polyamine for uptake assays and cotreated with indicated concentrations of MGBG or nonfluorescent polyamine for competition assays, with or without 90 min of 1-mM BV before treatment to inhibit polyamine uptake. To study endocytosis, cells were cultured in a 12-well plate at a density of 1 × 10^5^ cells per well. After 48 h, cells were treated with BV or endocytosis inhibitors (100-μM Dynasore, 50-μM genistein, and 50-μM Pitstop-2), while being starved for 30 min. Then, they were incubated at 4 °C for 15 min, treated with Alexa647-transferrin for 20 min, and incubated at 37 °C, 5% CO_2_ for 15 min. After incubating the cells with the treatments, they were washed with Dulbecco’s PBS (DPBS) modified with no calcium or magnesium (#D8537, Sigma) and collected using TrypLE Express (#12604021, Thermo Fisher) or trypsin in case of Alexa647-transferrin experiments. Cells were resuspended in DPBS supplemented with 1% bovine serum albumin (BSA; #3854.3, Carl Roth), and uptake was measured using an Attune NxT Flow Cytometer (Life Technologies). The settings were set at 1 × 10^4^ events (debris free) per condition, and the values of the mean fluorescent intensity of the blue laser channel (BL1; excitation = 488 nm, emission = 530 nm), with BODIPY–PUT/SPD/SPM, and the red laser channel (RL1; excitation = 637 nm, emission = 670 nm), with Alexa647-transferrin, were used for further analysis.

### Confocal microscopy

Cells were seeded in 12-well plates at a density of 5 × 10^3^ cells per well on coverslips, and they were left to grow for 2 days after the seeding. They were then treated with BODIPY–PUT for 90 min at 37 °C with or without 30-min pretreatment of 1-mM BV. Afterward, cells were washed with DPBS, fixed with 4% paraformaldehyde for 30 min at 37 °C, and washed again with DPBS. This was followed by either storing the cells in DPBS at 4 °C or directly proceeding further. First, cells were permeabilized for 30 min with 0.1% Triton X prepared in DPBS containing 0.1% Tween 20 (DPBS-T). Then, they were blocked for 60 min with 0.1 M glycine in DPBS-T and blocked further for 60 min with DPBS-T containing 10% FBS and 1% BSA. Subsequently, cells were incubated overnight at 4 °C with specific primary antibodies for EEA1, RAB11, RAB7, and LAMP1 (1:200 in DPBS-T with 1% FBS and 0.1% BSA). Then, cells were briefly blocked again for 15 min with DPBS-T containing 10% FBS and 1% BSA. Immunostaining was carried out with Alexa Fluor 594 goat anti-rabbit antibody (1:1000 in DPBS-T with 1% FBS and 0.1% BSA) for 60 min followed by nuclear staining using 4',6-diamidino-2-phenylindole (DAPI; #D9542, Sigma; 200 ng/ml in DPBS-T with 1% FBS and 0.1% BSA) for 15 min. Eventually, cells were washed thoroughly with DPBS-T, and coverslips were glued on glass slides using FluorSave reagent (#345789–20 ml, Millipore), left to dry, and then stored at −20 °C till imaging. Images were acquired using an LSM780 confocal microscope (Zeiss) using a 63x objective lens. Analyses for mean fluorescent intensity and Pearson’s coefficient for colocalization were performed using Fiji/ImageJ.

### Primer design, RNA extraction, and quantitative RT PCR

Primers were designed using Primer-BLAST or Primer3. BLAT was used to identify the target chromosome of the primers, exon-spanning was checked using Ensembl, and primers were synthesized by Integrated DNA Technologies. Primer sequences are displayed in Table 1. FKBP prolyl isomerase 1A (*FKBP1A*) was used as a reference gene (53). Standard curves from 5-fold serial dilutions of cDNA were used to determine amplification efficiencies. Primer specificity and validation were verified by analyzing the PCR product on an agarose gel in comparison with the Low Molecular Weight DNA Ladder (N3233S, New England Biolabs).

**Table 1:** Gene accession numbers and primer sequences

Gene	NCBI accession number	Primer sequence
*ATP13A1*	XM_007638292.1	TCATCCTCACCTCGGTTGTA
		ACACTGGGGTCACCTCTTTC
*ATP13A2*	XM_016963097.1	GCAGAAGACACATGGGAGGAC
		CACCAGTAGCTGCAGGTAGGA
*ATP13A3*	XM_007652544.2	GGTGGCAACTCATAGTACCG
		ACTGCATCACAAGGCATGA
*ATP13A4*	XM_007648363.1	CCAGAAAGTCTGGGATGGTT
		AAGTCCCTTCTCTGCCGTTA
*FKBP1A*^a^	XM_003499952.2	CTCTCGGGACAGAAACAAGC
		GACCTACACTCATCTGGGCTAC

a Primer sequence was taken from (53).

RNA was isolated from 5 × 10^6^ cells per cell line using NucleoSpin RNA plus Kit (#740984, Macherey-Nagel). RNA from the hamsters’ brains was extracted using TRIzol reagent (#15596026 & 15596018, Invitrogen) following the manufacturer’s instructions. The concentration and purity (260:230 nm and 260:280 nm absorbance ratios) were measured using a Nanodrop spectrophotometer (Thermo Fisher).

Conversion of 1 μg of RNA to cDNA was performed using RevertAid H Minus First Strand cDNA Synthesis Kit (#K1631, Thermo Fisher). Five-fold serial dilutions of cDNA were checked in duplicates. Reaction mixtures contained 12.5-μl SYBR Green master mix (#04707516001, Roche), 1-μl forward primer, 1-μl reverse primer, 5.5-μl distilled water, and 5-μl cDNA, whereas the negative controls were prepared using an equivalent volume of distilled water instead of cDNA. Reactions were performed in a 96-well plate using LightCycler machine (Roche), and amplification conditions were as follows: 95 °C for 10 min followed by 50 cycles at 95 °C for 10 s and 55 °C for 30 s, and the reaction was terminated at 95 °C for 1 min and then at 55 °C for 1 min followed by melting curve analysis from 55 to 95 °C. The mean Cq values were analyzed.

### Immunoblotting

Cells were detached using TrypLE Express, and then they were centrifuged at 300*g* for 5 min and washed once with DPBS and then centrifuged again. Total cell lysates were obtained by lysing the final pellets in RIPA buffer (#89900, Thermo Fisher) containing 10x protease (#S8830, Sigma) and 1x phosphatase (#A32957, Thermo Fisher) inhibitors. Protein concentration was determined using Pierce BCA Protein Assay Kit (#23227, Thermo Fisher) following the manufacturer’s instructions. Immunoblotting was performed as described (47). Primary antibodies against ATP13A2 (1:1000), ATP13A3 (1:1000), and GAPDH (1:5000) were incubated overnight. Anti-mouse IgG and anti-rabbit IgG horseradish peroxidase–linked secondary antibodies were used for 1 h at a dilution of 1:2000. Detection was performed using SuperSignal West Pico PLUS Chemiluminescent Substrate (#34580, Thermo Fisher) or SuperSignal West Femto Maximum Sensitivity Substrate (#34095, Thermo Fisher), and visualization was carried out using the Bio-Rad ChemiDoc MP imaging system.

### Synthesis of BODIPY-conjugated PUT

All starting materials and solvents were bought from commercial vendors and used without further purification. Reactions were analyzed by TLC on precoated 0.20-mm-thick ALUGRAM TLC sheets with fluorescent indicator and by LC-MS performed on a Prominence Ultrafast Liquid Chromatography system (Shimadzu) equipped with a 2 × 150 mm C18 analytical column (Waters X-Bridge) coupled to an MS-2020 single quadrupole mass analyzer (Shimadzu). A linear gradient of 5 to 80% acetonitrile in water (with 0.1% formic acid [FA]) was utilized. Silica column chromatography was performed using 230 to 400 mesh silica (Kieselgel 60). NMR spectra were recorded in CDCl_3_ and measured using a Bruker Ultrashield 400 MHz NMR Spectrometer. Chemical shifts are reported in ppm relative to the residual solvent peak.

#### Tert-butyl (4-aminobutyl)carbamate (1)

**Figure:**
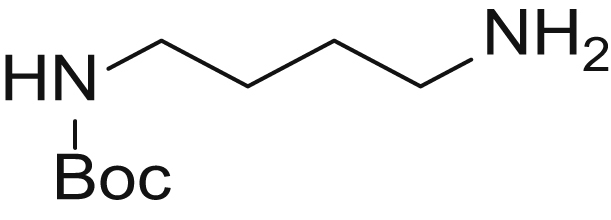

Boc-protected PUT **1** was synthesized based on the method of Dardonville *et al.* (54). To this end, PUT (323 mg, 4 eq., 3.67 mmol) was dissolved in 4-ml CHCl_3_ and cooled to 0 °C. Boc_2_O (526 μl, 1eq., 2.29 mmol) was dissolved in 10-ml CHCl_3_ and slowly added to the PUT solution over the course of 2 h. The reaction mixture was then allowed to warm to RT and stirred overnight. Upon completion of the reaction (TLC: PE/EtOAC 1:1), the solvents were evaporated and the crude product was redissolved in 20-ml dichloromethane (DCM). The crude mixture was extracted three times with water. The organic fraction was collected, dried over MgSO_4_, and concentrated. The product was further purified using column chromatography (petroleum ether/EA 1:1 → EA/MeOH/TEA 9:1:0.01) to obtain the title compound as a colorless oil. (169 mg, yield = 98%) electrospray ionization-mass spectrometry (ESI-MS): *m/z* calculated for C_9_H_20_N_2_O_2_ [M + H]^+^ requires 189.15, found 188.95

#### 3-Azidopropyl 4-methylbenzenesulfonate (2)

**Figure:**
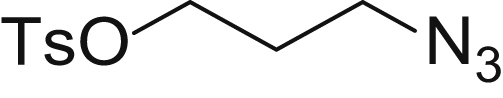

Tosylate **2** was synthesized based on the procedure of Chan *et al.* (55). To this end, 3-chloropropanol (1.00 g, 10.58 mmol, 1 eq.) was dissolved in 10-ml water. NaN_3_ (688 mg, 10.58 mmol, 1 eq.) was added to this solution, and the reaction was refluxed overnight. Upon completion of the reaction (petroleum ether/EA 1:1), the reaction mixture was cooled to RT and extracted three times with 20-ml DCM. The organic fractions were collected, dried over MgSO_4_, and evaporated to dryness. The crude product was redissolved in 15-ml dry DCM. Triethylamine (2.95 ml, 2 eq., 21.17 mmol) was added, and the mixture was cooled to 0 °C. Tosyl chloride (2.44 g, 1.2 eq., 11.64 mmol) was added to the reaction mixture, which was subsequently allowed to warm to RT and stirred overnight. Upon completion of the reaction (TLC: PE:EtOAc 4:1), the solvents were evaporated and the crude product purified by column chromatography (PE:EtOAC 1:0 → 9:1) to obtain the title compound as a colorless solid. (1.554 g, yield = 58% over two steps) ESI-MS: *m/z* calculated for C_10_H_13_N_3_O_3_S [M + H]^+^ requires 256.07, found 256.90.

#### Tert-butyl (4-((3-azidopropyl)amino)butyl)carbamate (3)

**Figure:**
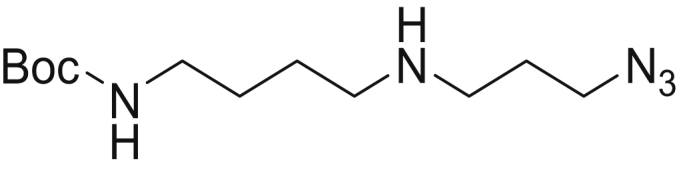

Boc-PUT **1** (88 mg, 467 μmol, 1 eq.) was dissolved in 2-ml dry tetrahydrofuran under argon atmosphere. Diisopropylethylamine (333 μl, 1.87 mmol, 4 eq.) was added to this solution, which was subsequently cooled to 0 °C. Tosylate **2** was added gradually to the reaction, which was subsequently allowed to warm up to RT. The reaction mixture was stirred overnight at RT. Upon completion of the reaction (TLC: DCM/MeOH 9:1), the solvents were evaporated and the crude product purified over column chromatography (DCM/MeOH 9:1) to obtain the title compound as a colorless oil. (38.37 mg yield = 30%) ESI-MS: *m/z* calculated for C_12_H_25_N_5_O_2_ [M + H]^+^ requires 272.20, found 272.00.

#### 5,5-Difluoro-1,3,7,9-tetramethyl-10-(pent-4-yn-1-yl)-9a,10-dihydro-5H-5l4,6l4-dipyrrolo[1,2-c:2',1'-f][1,3,2]diazaborinine (4)

**Figure:**
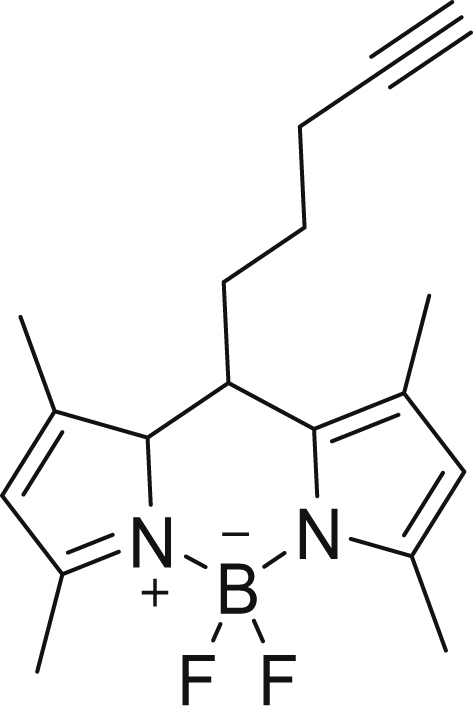

Alkyne–BODIPY **4** was synthesized according to the method described in Vanhoutte *et al.* (30). The identity was in agreement with the data reported in the literature. ESI-MS: *m/z* calculated for C_18_H_21_BF_2_N_2_ [M + H]^+^ 315.18, found 315.00

#### Tert-butyl (4-((3-(4-(3-(5,5-difluoro-1,3,7,9-tetramethyl-5H-4l4,5l4-dipyrrolo[1,2-c:2',1'-f][1,3,2]diazaborinin-10-yl)propyl)-1H-1,2,3-triazol-1-yl)propyl)amino)butyl)carbamate (5)

**Figure:**
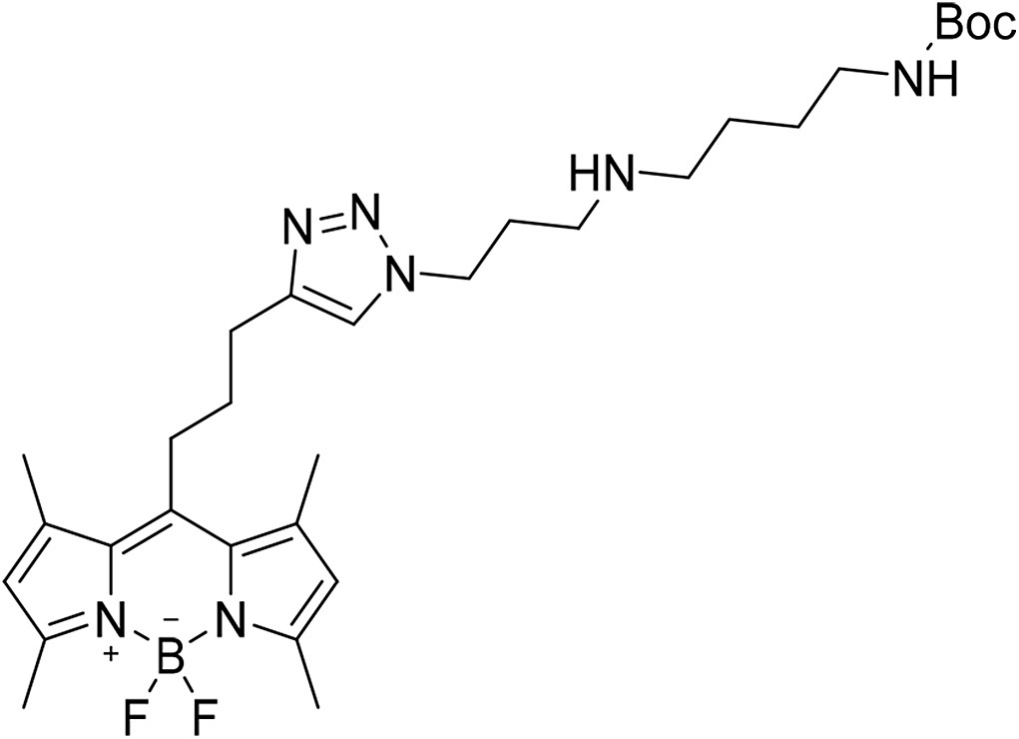

Compound **3** (24 mg, 1 eq., 88.44 μmol) was dissolved in 2-ml DCM. Alkyne–BODIPY **4** (29.18 mg, 1.05 eq., 92.86 μmol), diisopropylethylamine (63 μl, 4 eq., 353.77 μmol), and subsequently CuBr (6.34 mg, 0.5 eq., 44.22 μmol) were added to the reaction mixture. The reaction was stirred at RT for 3 h. Upon completion of the reaction (TLC: DCM/MeOH 9:1), the solvents were evaporated and the crude product purified using column chromatography (DCM/MeOH 9:1) to obtain the title compound as a red oil. (32.45 mg, yield = 63%) ESI-MS: *m/z* calculated for C_30_H_46_BF_2_N_7_O_2_ [M + H]^+^ requires 586.37, found 586.20.

#### N1-(3-(4-(3-(5,5-difluoro-1,3,7,9-tetramethyl-5H-4l4,5l4-dipyrrolo[1,2-c:2',1'-f][1,3,2]diazaborinin-10-yl)propyl)-1H-1,2,3-triazol-1-yl)propyl)butane-1,4-diamine dihydrochloride (6)

**Figure:**
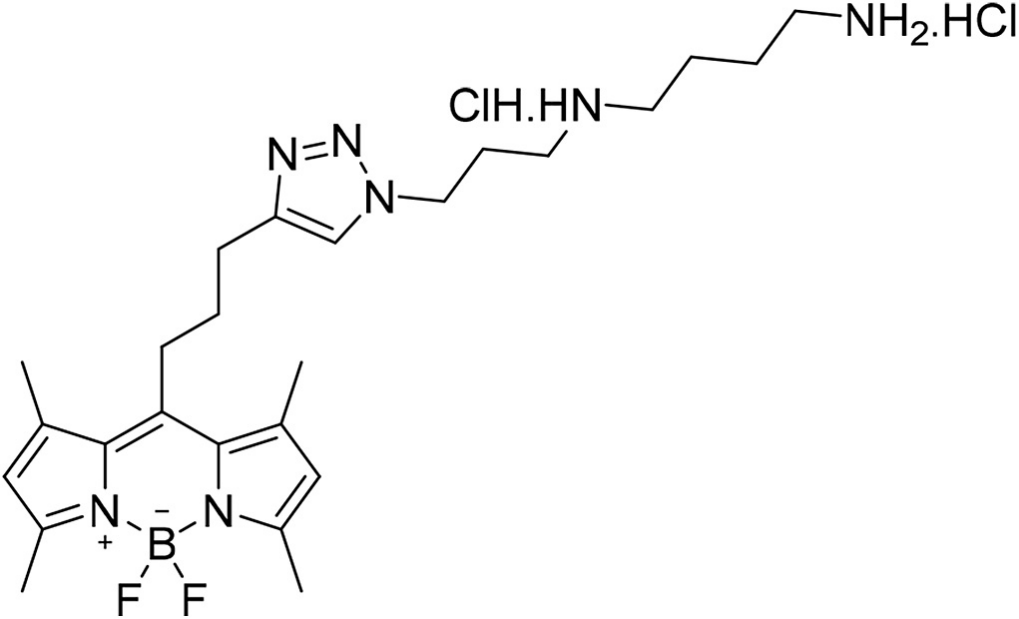

Compound **5** (32.45 mg, 55.42 μmol) was dissolved in 6-ml 4N HCl in dioxane and stirred for 5 min at RT. Upon completion of the reaction, the solvents were evaporated to obtain the title compound as a red solid (30.94 mg, yield = 100%). ESI-MS: *m/z* calculated for C_25_H_40_BCl_2_F_2_N_7_ [M+H-HCl]^+^ requires 486.32, found 486.15. ^1^H NMR (400 MHz, CDCl3, 298K): δ = 7.91 to 7.48 (m, 2H), 7.24 to 6.94 (m, 1H), 6.44 to 5.66 (m, 2H), 3.83 to 3.53 (m, 4H), 3.22 to 3.00 (m, 4H), 2.71 to 1.81 (m, 8H), 1.63 to 1.32 (m, 14H).

### Proteomics

#### Sample preparation and LC-MS/MS analysis

A count of 5 × 10^6^ trypsinized cells per cell line was collected by two centrifugation spins at 300*g* for 5 min, followed by pellet resuspension in DPBS. Dry pellets were snap-frozen in liquid nitrogen and stored at −80 °C. Proteomics was performed on four biological replicates of each cell line.

A total of 8 membrane fraction samples were prepared for LC-MS/MS analysis. Sample preparation was based on methods reported earlier by Ning *et al.* in 2013 and 2014 (56, 57). For this purpose, 500 μl of a 100-mM Hepes solution was added to samples containing 100-μg protein in 500-μl 0.25 M sucrose and protease inhibitors to reach a final Hepes concentration of 50 mM. Amphipol (APols) A8-35 (Anatrace Products) was added to a final concentration of 1%. Samples were homogenized on ice by using a tip sonicator (VC100 Vibra-Cell, Sonics & Materials) using the following settings: 40-Hz amplitude, 5 s ON, 2 s OFF, 3 cycles, 7 to 9 W output. After sonication, samples were incubated at RT with shaking (650 rpm) for 10 min. Reduction of sample protein disulfide bonds was achieved by adding DTT (dithiothreitol, Sigma Aldrich) to a final concentration of 5 mM and incubation for 30 min at 50 °C and 650 rpm. Alkylation was performed by adding iodoacetamide (Fluka) to a final concentration of 10 mM and incubation at RT in the dark for 15 min at 650 rpm. After reduction and alkylation, the pH was adjusted with 10% formic acid (FA; Acros Organics) to a final concentration of 1% FA and samples incubated for 10 min at RT and 500 rpm. APols were precipitated by centrifugation at 16,000*g* for 5 min at RT and the supernatant discarded. The APols pellet was washed with 0.1% FA for 5 min at 500 rpm and precipitated again by centrifugation at 16,000*g* for 5 min at RT. The supernatant was again discarded, and the pellet was solubilized in 200 μl of 100-mM tetraethylammonium bicarbonate (Sigma Aldrich). One microgram of trypsin was added to each sample, and digestion was performed overnight at 37 °C and 800 rpm shaking. Samples were briefly spun down, and the pH adjusted to a final FA concentration of 1% and incubated for 10 min at RT at 650 rpm. APols were precipitated by centrifugation at 16,000*g* for 10 min at RT, and the supernatant, containing the tryptic peptides, was transferred to MS vials and vacuum-dried completely. Samples were stored at −20 °C until measured by LC-MS/MS.

Purified peptides were redissolved in a 30-μl loading solvent (0.1% trifluoroacetic acid in water/acetonitrile (96:4, v/v)), and the peptide concentration was determined by measuring on a Lunatic spectrophotometer (Unchained Labs) (Mendes Maia *et al.* 2020 ACS Omega, in press). 2 μg of peptide material of each sample was injected for LC-MS/MS analysis on an Ultimate 3000 RSLC nano-LC (Thermo Fisher Scientific, Bremen, Germany) in-line connected to a Q Exactive HF mass spectrometer (Thermo Fisher Scientific) equipped with a nanospray flex ion source (Thermo Fisher Scientific).

Trapping was performed at 10 μl/min for 4 min in loading solvent A on a 20-mm trapping column (made in-house, 100-μm internal diameter, 5-μm beads, C18 Reprosil-HD, Dr Maisch, Germany). Peptide separation after trapping was performed on a 200-cm-long micropillar array column (PharmaFluidics) with C18-endcapped functionality. The Ultimate 3000’s column oven was set to 50 °C. For proper ionization, a fused silica PicoTip emitter (10-μm inner diameter) (New Objective) was connected to the μPAC outlet union and a grounded connection was provided to this union. Peptides were eluted by a nonlinear gradient from 1 to 55% MS solvent B (0.1% FA in water/acetonitrile (2:8, v/v)) over 145 min, starting at a flow rate of 750 nl/min switching to 300 nl/min after 15 min, followed by a 15-min washing phase plateauing at 99% MS solvent B. Re-equilibration with 99% MS solvent A (0.1% FA in water) was performed at 300 nl/min for 45 min followed by 5 min at 750 nl/min adding up to a total run length of 210 min. The mass spectrometer was operated in a data-dependent, positive ionization mode, automatically switching between MS and MS/MS acquisition for the 16 most abundant peaks in a given MS spectrum. The source voltage was 2.2 kV, and the capillary temperature was 275 °C. One MS1 scan (m/z 375–1,500, AGC target 3 × 10^6^ ions, maximum ion injection time 60 ms), acquired at a resolution of 60,000 (at 200 m/z), was followed by up to 16 tandem MS scans (resolution 15,000 at 200 m/z) of the most intense ions fulfilling predefined selection criteria (AGC target 1 × 10^5^ ions, maximum ion injection time 80 ms, isolation window 1.5 Da, fixed first mass 145 m/z, spectrum data type: centroid, intensity threshold 1.3 × 10^4^, exclusion of unassigned, 1, 7, 8, >8 positively charged precursors, peptide match preferred, exclude isotopes on, dynamic exclusion time 12 s). The higher-energy collisional dissociation was set to 28% normalized collision energy, and the polydimethylcyclosiloxane background ion at 445.12003 Da was used for internal calibration (lock mass).

#### Data analysis

Data analysis was performed with MaxQuant (version 1.6.9.0) using the Andromeda search engine with default search settings including a false discovery rate (FDR) set at 1% on both the peptide and protein level (58, 59). Spectra were searched against the Chinese hamster (CHO K1 cell line) Swiss-Prot database (from September 2019 with 23,889 entries) and NCBI single protein databases for hamster ATP13A1, ATP13A2, ATP13A3, and ATP13A4. The mass tolerance for precursor and fragment ions was set to 4.5 and 20 ppm, respectively, during the main search. Enzyme specificity was set to the C-terminal of arginine and lysine, also allowing cleavage next to prolines with a maximum of two missed cleavages. Variable modifications were set to oxidation of methionine residues and acetylation of protein N-termini. Matching between runs was enabled with a matching time window of 0.7 min and an alignment time window of 20 min. Only proteins with at least one unique or razor peptide were retained, leading to the identification of 5040 proteins. Proteins were quantified by the MaxLFQ algorithm integrated into the MaxQuant software. A minimum ratio count of two unique or razor peptides was required for quantification. Further data analysis was performed with the Perseus software (version 1.6.7.0) after uploading the protein groups file from MaxQuant (60). Reverse database hits were removed, and replicate samples were grouped. Proteins with less than three valid values in at least one group were removed, and missing values were imputed from a normal distribution around the detection limit resulting in 3382 quantified proteins, which were subsequently used for further data analysis. These quantified proteins were subjected to a two-sided, unpaired t-test using permutation-based multiparameter correction with 1000 randomizations and an FDR of 1%. The results of this t-test are shown in the volcano plot in Fig. S2*B*. For each protein, the log2 (MG/WT) fold change value is indicated on the X-axis, whereas the statistical significance (-log *p*-value) is indicated on the Y-axis. Proteins outside the curved lines, set by an FDR value of 0.01 and an S0 value of 0.1 in the Perseus software, represent specific significant upregulated or downregulated proteins.

### Genomics

Samples were prepared with the KAPA Library HyperPrep Kit (#07962363001, Roche) according to the manufacturer protocol. Samples were indexed to allow for multiplexing and subsequently sequenced on an Illumina NovaSeq platform according to the manufacturer recommendations. Paired-end reads of 150-bp length were produced with a total of 59 M reads. Quality control of raw reads was performed with FastQC, v0.11.7 (61). Alignment was performed with Burrows–Wheeler aligner maximum exact matches software (62) against the Chinese Hamster reference genome. Reads mapping to multiple loci in the reference genome were discarded. Resulting binary alignment map files were handled with SAMtools, v1.5 (63). Validation of binary alignment map files and duplicate marking was performed with Picard. Variants and VCF files were produced with GATK 3.7 (64). The effect of variants was determined with Ensembl Variant Effect Predictor (65).

### Statistical analysis

Data are presented as the mean ± SE or mean ± SD, and ‘*n*’ indicates the number of independent experiments. Statistical analysis was conducted by one-/two-way ANOVA with Bonferroni *post hoc* corrections or unpaired (Mann-Whitney) t-test (∗/€*p* < 0.05, ∗∗/€€*p* < 0.01, ^∗∗∗/^^€€€^*p* < 0.001, and ^∗∗∗∗/^^€€€€^*p* < 0.0001).

## Data availability

The mass spectrometry proteomics data have been deposited to the ProteomeXchange Consortium *via* the PRIDE (66) partner repository (http://www.ebi.ac.uk/pride) with the data set identifier PXD020559 and 10.6019/PXD020559. And the full genomics data set can be accessed on GenBank (sequence read archive accession: PRJNA627458) *via* the link https://www.ncbi.nlm.nih.gov/sra/PRJNA627458. All of the remaining data are contained in the article or its supporting information.

## Conflict of interest

The authors declare that they have no conflicts of interest with the contents of this article.

## Supporting information

Figures S1–S6

JBC_JBC-2020-013908_10

## References

[1] Pegg A.E. (2016). Functions of polyamines in mammals. J. Biol. Chem..

[2] Pegg A.E. (2009). Mammalian polyamine metabolism and function. IUBMB Life.

[3] Eisenberg T., Abdellatif M., Schroeder S., Primessnig U., Stekovic S., Pendl T., Harger A., Schipke J., Zimmermann A., Schmidt A., Tong M., Ruckenstuhl C., Dammbrueck C., Gross A.S., Herbst V. (2016). Cardioprotection and lifespan extension by the natural polyamine spermidine. Nat. Med..

[4] Eisenberg T., Knauer H., Schauer A., Büttner S., Ruckenstuhl C., Carmona-Gutierrez D., Ring J., Schroeder S., Magnes C., Antonacci L., Fussi H., Deszcz L., Hartl R., Schraml E., Criollo A. (2009). Induction of autophagy by spermidine promotes longevity. Nat. Cell Biol..

[5] Pegg A.E. (2013). Toxicity of polyamines and their metabolic products. Chem. Res. Toxicol..

[6] Miller-Fleming L., Olin-Sandoval V., Campbell K., Ralser M. (2015). Remaining mysteries of molecular biology: the role of polyamines in the cell. J. Mol. Biol..

[7] Heaton M.A., Flintoff W.F. (1988). Methylglyoxal-bis(guanylhydrazone)-resistant Chinese hamster ovary cells: genetic evidence that more than a single locus controls uptake. J. Cell Physiol..

[8] Persson L., Holm I., Ask A., Heby O. (1988). Curative effect of DL-2-difluoromethylornithine on mice bearing mutant L1210 leukemia cells deficient in polyamine uptake. Cancer Res..

[9] Byers T.L., Wechter R., Nuttall M.E., Pegg A.E. (1989). Expression of a human gene for polyamine transport in Chinese-hamster ovary cells. Biochem. J..

[10] Evageliou N.F., Haber M., Vu A., Laetsch T.W., Murray J., Gamble L.D., Cheng N.C., Liu K., Reese M., Corrigan K.A., Ziegler D.S., Webber H., Hayes C.S., Pawel B., Marshall G.M. (2016). Polyamine antagonist therapies inhibit neuroblastoma initiation and progression. Clin. Cancer Res..

[11] Delcros J.-G., Tomasi S., Carrington S., Martin B., Renault J., Blagbrough I.S., Uriac P. (2002). Effect of spermine conjugation on the cytotoxicity and cellular transport of acridine. J. Med. Chem..

[12] Wang M., Phanstiel O., von Kalm L. (2017). Evaluation of polyamine transport inhibitors in a drosophila epithelial model suggests the existence of multiple transport systems. Med. Sci. (Basel).

[13] Mandel J.-L., Flintoff W.F. (1978). Isolation of mutant mammalian cells altered in polyamine transport. J. Cell Physiol..

[14] Byers T.L., Kameji R., Rannels D.E., Pegg A.E. (1987). Multiple pathways for uptake of paraquat, methylglyoxal bis(guanylhydrazone), and polyamines. Am. J. Physiol. Cell Physiol..

[15] Belting M., Mani K., Jönsson M., Cheng F., Sandgren S., Jonsson S., Ding K., Delcros J.-G., Fransson L.-Å. (2003). Glypican-1 is a vehicle for polyamine uptake in mammalian cells: a pivotal role for nitrosothiol-derived nitric oxide. J. Biol. Chem..

[16] Soulet D., Gagnon B., Rivest S., Audette M., Poulin R. (2004). A fluorescent probe of polyamine transport accumulates into intracellular acidic vesicles via a two-step mechanism. J. Biol. Chem..

[17] Sharpe J.G., Seidel E.R. (2005). Polyamines are absorbed through a y+ amino acid carrier in rat intestinal epithelial cells. Amino Acids.

[18] Soulet D., Covassin L., Kaouass M., Charest-Gaudreault R., Audette M., Poulin R. (2002). Role of endocytosis in the internalization of spermidine-C(2)-BODIPY, a highly fluorescent probe of polyamine transport. Biochem. J..

[19] Muth A., Madan M., Archer J.J., Ocampo N., Rodriguez L., Phanstiel O. (2014). Polyamine transport inhibitors: design, synthesis, and combination therapies with difluoromethylornithine. J. Med. Chem..

[20] Liao C.-P., Phanstiel O., Lasbury M.E., Zhang C., Shao S., Durant P.J., Cheng B.-H., Lee C.-H. (2009). Polyamine transport as a target for treatment of pneumocystis pneumonia. Antimicrob. Agents Chemother..

[21] Nazifi S.M., Sadeghi-Aliabadi H., Fassihi A., Saghaie L. (2019). Structure–activity relationship of polyamine conjugates for uptake via polyamine transport system. Struct. Chem..

[22] Phanstiel O., Archer J.J. (2012). Chapter 7 design of polyamine transport inhibitors as therapeutics. Polyamine Drug Discovery.

[23] Uemura T., Stringer D.E., Blohm-Mangone K.A., Gerner E.W. (2010). Polyamine transport is mediated by both endocytic and solute carrier transport mechanisms in the gastrointestinal tract. Am. J. Physiol. Gastrointest. Liver Physiol..

[24] Uemura T., Yerushalmi H.F., Tsaprailis G., Stringer D.E., Pastorian K.E., Hawel L., Byus C.V., Gerner E.W. (2008). Identification and characterization of a diamine exporter in colon epithelial cells. J. Biol. Chem..

[25] Ding K., Sandgren S., Mani K., Belting M., Fransson L.-Å. (2001). Modulations of glypican-1 heparan sulfate structure by inhibition of endogenous polyamine synthesis: mapping of spermine-binding sites and heparanase, heparin lyase, and nitric oxide/nitrite cleavage sites. J. Biol. Chem..

[26] Haysmark B., Fransson L.-Å., Jönsson M., Belting M., Persson S. (1996). Heparan sulphate/heparin glycosaminoglycans with strong affinity for the growth-promoter spermine have high antiproliferative activity. Glycobiology.

[27] Hiasa M., Miyaji T., Haruna Y., Takeuchi T., Harada Y., Moriyama S., Yamamoto A., Omote H., Moriyama Y. (2014). Identification of a mammalian vesicular polyamine transporter. Sci. Rep..

[28] van Veen S., Martin S., Van den Haute C., Benoy V., Lyons J., Vanhoutte R., Kahler J.P., Decuypere J.-P., Gelders G., Lambie E., Zielich J., Swinnen J.V., Annaert W., Agostinis P., Ghesquière B. (2020). ATP13A2 deficiency disrupts lysosomal polyamine export. Nature.

[29] Sørensen D.M., Holemans T., van Veen S., Martin S., Arslan T., Haagendahl I.W., Holen H.W., Hamouda N.N., Eggermont J., Palmgren M., Vangheluwe P. (2018). Parkinson disease related ATP13A2 evolved early in animal evolution. PLoS One.

[30] Vanhoutte R., Kahler J.P., Martin S., van Veen S., Verhelst S.H.L. (2018). Clickable polyamine derivatives as chemical probes for the polyamine transport system. ChemBioChem.

[31] Saunders N.A., Ilett K.F., Minchin R.F. (1989). Pulmonary alveolar macrophages express a polyamine transport system. J. Cell Physiol..

[32] Hammond S., Kaplarevic M., Borth N., Betenbaugh M.J., Lee K.H. (2012). Chinese hamster genome database: an online resource for the CHO community at www.CHOgenome.org. Biotechnol. Bioeng..

[33] Schaum N., Karkanias J., Neff N.F., May A.P., Quake S.R., Wyss-Coray T., Darmanis S., Batson J., Botvinnik O., Chen M.B., Chen S., Green F., Jones R.C., Maynard A., Penland L. (2018). Single-cell transcriptomics of 20 mouse organs creates a Tabula Muris. Nature.

[34] Uhlén M., Fagerberg L., Hallström B.M., Lindskog C., Oksvold P., Mardinoglu A., Sivertsson Å., Kampf C., Sjöstedt E., Asplund A., Olsson I., Edlund K., Lundberg E., Navani S., Szigyarto C.A.-K. (2015). Proteomics. Tissue-based map of the human proteome. Science.

[35] Mayle K.M., Le A.M., Kamei D.T. (2012). The intracellular trafficking pathway of transferrin. Biochim. Biophys. Acta..

[36] van Veen S., Sørensen D.M., Holemans T., Holen H.W., Palmgren M.G., Vangheluwe P. (2014). Cellular function and pathological role of ATP13A2 and related P-type transport ATPases in Parkinson's disease and other neurological disorders. Front. Mol. Neurosci..

[37] Loizides-Mangold U. (2005). The antizyme family: polyamines and beyond. IUBMB Life.

[38] Gamble L.D., Purgato S., Murray J., Xiao L., Yu D.M.T., Hanssen K.M., Giorgi F.M., Carter D.R., Gifford A.J., Valli E., Milazzo G., Kamili A., Mayoh C., Liu B., Eden G. (2019). Inhibition of polyamine synthesis and uptake reduces tumor progression and prolongs survival in mouse models of neuroblastoma. Sci. Transl. Med..

[39] Madan M., Patel A., Skruber K., Geerts D., Altomare D.A., Iv O.P. (2016). ATP13A3 and caveolin-1 as potential biomarkers for difluoromethylornithine-based therapies in pancreatic cancers. Am. J. Cancer Res..

[40] Gräf S., Haimel M., Bleda M., Hadinnapola C., Southgate L., Li W., Hodgson J., Liu B., Salmon R.M., Southwood M., Machado R.D., Martin J.M., Treacy C.M., Yates K., Daugherty L.C. (2018). Identification of rare sequence variation underlying heritable pulmonary arterial hypertension. Nat. Commun..

[41] Barozzi C., Galletti M., Tomasi L., De Fanti S., Palazzini M., Manes A., Sazzini M., Galiè N. (2019). A combined targeted and whole exome sequencing approach identified novel candidate genes involved in heritable pulmonary arterial hypertension. Sci. Rep..

[42] Morrell N.W., Aldred M.A., Chung W.K., Elliott C.G., Nichols W.C., Soubrier F., Trembath R.C., Loyd J.E. (2019). Genetics and genomics of pulmonary arterial hypertension. Eur. Respir. J..

[43] Di Fonzo A., Chien H.F., Socal M., Giraudo S., Tassorelli C., Iliceto G., Fabbrini G., Marconi R., Fincati E., Abbruzzese G., Marini P., Squitieri F., Horstink M.W., Montagna P., Libera A.D. (2007). ATP13A2 missense mutations in juvenile parkinsonism and young onset Parkinson disease. Neurology.

[44] Ramirez A., Heimbach A., Gründemann J., Stiller B., Hampshire D., Cid L.P., Goebel I., Mubaidin A.F., Wriekat A.-L., Roeper J., Al-Din A., Hillmer A.M., Karsak M., Liss B., Woods C.G. (2006). Hereditary parkinsonism with dementia is caused by mutations in ATP13A2, encoding a lysosomal type 5 P-type ATPase. Nat. Genet..

[45] Holemans T., Sørensen D.M., van Veen S., Martin S., Hermans D., Kemmer G.C., Van den Haute C., Baekelandt V., Günther Pomorski T., Agostinis P., Wuytack F., Palmgren M., Eggermont J., Vangheluwe P. (2015). A lipid switch unlocks Parkinson's disease-associated ATP13A2. Proc. Natl. Acad. Sci. U. S. A..

[46] Gitler A.D., Chesi A., Geddie M.L., Strathearn K.E., Hamamichi S., Hill K.J., Caldwell K.A., Caldwell G.A., Cooper A.A., Rochet J.-C., Lindquist S. (2009). Alpha-synuclein is part of a diverse and highly conserved interaction network that includes PARK9 and manganese toxicity. Nat. Genet..

[47] Martin S., van Veen S., Holemans T., Demirsoy S., van den Haute C., Baekelandt V., Agostinis P., Eggermont J., Vangheluwe P. (2016). Protection against mitochondrial and metal toxicity depends on functional lipid binding sites in ATP13A2. Parkinsons Dis..

[48] Rinaldi D.E., Corradi G.R., Cuesta L.M., Adamo H.P., de Tezanos Pinto F. (2015). The Parkinson-associated human P5B-ATPase ATP13A2 protects against the iron-induced cytotoxicity. Biochim. Biophys. Acta..

[49] Schmidt K., Wolfe D.M., Stiller B., Pearce D.A. (2009). Cd2+, Mn2+, Ni2+ and Se2+ toxicity to Saccharomyces cerevisiae lacking YPK9p the orthologue of human ATP13A2. Biochem. Biophys. Res. Commun..

[50] Das K.C., Misra H.P. (2004). Hydroxyl radical scavenging and singlet oxygen quenching properties of polyamines. Mol. Cell Biochem..

[51] LØVaas E., Sies H. (1996). Antioxidative and metal-chelating effects of polyamines. Advances in Pharmacology.

[52] Osório L., Gijsbers R., Oliveras-Salvá M., Michiels A., Debyser Z., Van den Haute C., Baekelandt V. (2014). Viral vectors expressing a single microRNA-based short-hairpin RNA result in potent gene silencing *in vitro* and *in vivo*. J. Biotechnol..

[53] Brown A.J., Gibson S., Hatton D., James D.C. (2018). Transcriptome-based identification of the optimal reference CHO genes for normalisation of qPCR data. Biotechnol. J..

[54] Dardonville C., Fernandez-Fernandez C., Gibbons S.-L., Ryan G.J., Jagerovic N., Gabilondo A.M., Meana J.J., Callado L.F., Callado L.F. (2006). Synthesis and pharmacological studies of new hybrid derivatives of fentanyl active at the mu-opioid receptor and I2-imidazoline binding sites. Bioorg. Med. Chem..

[55] Chan E.W.C., Baek P., Barker D., Travas-Sejdic J. (2015). Highly functionalisable polythiophene phenylenes. Polym. Chem..

[56] Ning Z., Hawley B., Seebun D., Figeys D. (2014). APols-aided protein precipitation: a rapid method for concentrating proteins for proteomic analysis. J. Membr. Biol..

[57] Ning Z., Seebun D., Hawley B., Chiang C.-K., Figeys D. (2013). From cells to peptides: “one-stop” integrated proteomic processing using amphipols. J. Proteome Res..

[58] Cox J., Hein M.Y., Luber C.A., Paron I., Nagaraj N., Mann M. (2014). Accurate proteome-wide label-free quantification by delayed normalization and maximal peptide ratio extraction, termed MaxLFQ. Mol. Cell Proteomics.

[59] Cox J., Mann M. (2008). MaxQuant enables high peptide identification rates, individualized p.p.b.-range mass accuracies and proteome-wide protein quantification. Nat. Biotechnol..

[60] Tyanova S., Temu T., Sinitcyn P., Carlson A., Hein M.Y., Geiger T., Mann M., Cox J. (2016). The Perseus computational platform for comprehensive analysis of (prote)omics data. Nat. Methods.

[61] Andrews S. (Babraham Bioinformatics, UK: 2010.). FastQC: A Quality Control Tool for High Throughput Sequence Data.

[62] Li H., Durbin R. (2009). Fast and accurate short read alignment with Burrows-Wheeler transform. Bioinformatics.

[63] Li H., Handsaker B., Wysoker A., Fennell T., Ruan J., Homer N., Marth G., Abecasis G., Durbin R., 1000 Genome Project Data Processing Subgroup (2009). The sequence alignment/map format and SAMtools. Bioinformatics.

[64] Poplin R., Ruano-Rubio V., DePristo M.A., Fennell T.J., Carneiro M.O., Van der Auwera G.A., Kling D.E., Gauthier L.D., Levy-Moonshine A., Roazen D., Shakir K., Thibault J., Chandran S., Whelan C., Lek M. (2018). Scaling accurate genetic variant discovery to tens of thousands of samples. bioRxiv.

[65] McLaren W., Gil L., Hunt S.E., Riat H.S., Ritchie G.R.S., Thormann A., Flicek P., Cunningham F. (2016). The Ensembl variant effect predictor. Genome Biol..

[66] Perez-Riverol Y., Csordas A., Bai J., Bernal-Llinares M., Hewapathirana S., Kundu D.J., Inuganti A., Griss J., Mayer G., Eisenacher M., Pérez E., Uszkoreit J., Pfeuffer J., Sachsenberg T., Yilmaz S. (2019). The PRIDE database and related tools and resources in 2019: improving support for quantification data. Nucleic Acids Res..

