# Supplementary material for: ATP13A3 is a major component of the enigmatic mammalian polyamine transport system
Source: J Biol Chem. 2020 Dec 17;296:100182. doi: 10.1074/jbc.RA120.013908 (PMC7948421; doi:10.1074/jbc.RA120.013908)
Supplement: JBC_JBC-2020-013908_10 [file mmc2.pdf]

ATP13A3 is a major component of the enigmatic mammalian polyamine transport system

Norin Nabil Hamouda[1], Chris Van den Haute[2,3], Roeland Vanhoutte[4], Ragna Sannerud[5], Mujahid Azfar[1], Rupert Mayer[6], Álvaro Cortés Calabuig[7], Johannes V. Swinnen[8], Patrizia Agostinis[9,10], Veerle Baekelandt[2], Wim Annaert[5], Francis Impens[6], Steven H. L.Verhelst[4,11], Jan Eggermont[1], Shaun Martin[1,#], Peter Vangheluwe[1,#*]

[1] Laboratory of Cellular Transport Systems, [4] Laboratory of Chemical Biology, [9] Laboratory of Cell Death Research & Therapy, Department of Cellular and Molecular Medicine, KU Leuven, Leuven, Belgium

[2] Laboratory for Neurobiology and Gene Therapy, [5] VIB-KU Leuven Laboratory of Membrane Trafficking; Department of Neurosciences, KU Leuven, Leuven, Belgium

[3] Leuven Viral Vector Core, KU Leuven, Leuven, Belgium

[6] VIB Center for Medical Biotechnology, VIB Proteomics Core, Department for Biomolecular Medicine, Ghent University, Ghent, Belgium

[7] Genomics Core Leuven, KU Leuven, Leuven, Belgium

[8] Laboratory of Lipid Metabolism and Cancer, Department of Oncology, LKI – Leuven Cancer Institute, KU Leuven, Leuven, Belgium

[10] VIB-KU Leuven Center for Cancer Biology, Department of Oncology, KU Leuven, Leuven, Belgium

[11] Leibniz Institute for Analytical Sciences ISAS, Otto-Hahn-Str. 6b, 44227 Dortmund, Germany

[#] Joint last authors

*** Corresponding author:**
Prof. Dr. Peter Vangheluwe
Department of Cellular and Molecular Medicine
KU Leuven Campus Gasthuisberg, O&N I Herestraat 49 - box 802, 3000 Leuven
Tel.: ±32 16 33 07 20
E-mail: peter.vangheluwe@kuleuven.be
ORCID ID: https://orcid.org/0000-0002-7822-2944

Running Title:
ATP13A3 is involved in Polyamine Transport

List of contents

Supporting Information Figures

- Figure S1. Polyamine biosynthesis pathway and its inhibitors.
- Figure S2. Effect of polyamines on viability of CHO-MG cells.
- Figure S3. ATP13A3 is downregulated in CHO-MG cells.
- Figure S4. Uptake of BODIPY-PUT uptake falls within the linear phase.
- Figure S5. Competition of MGBG with SPD and SPM in CHO-WT cells.
- Figure S6. Expression of wild-type ATP13A2 restores polyamine uptake, but it does not affect MGBG resistance in CHO-MG cells.

Supporting Information Figures

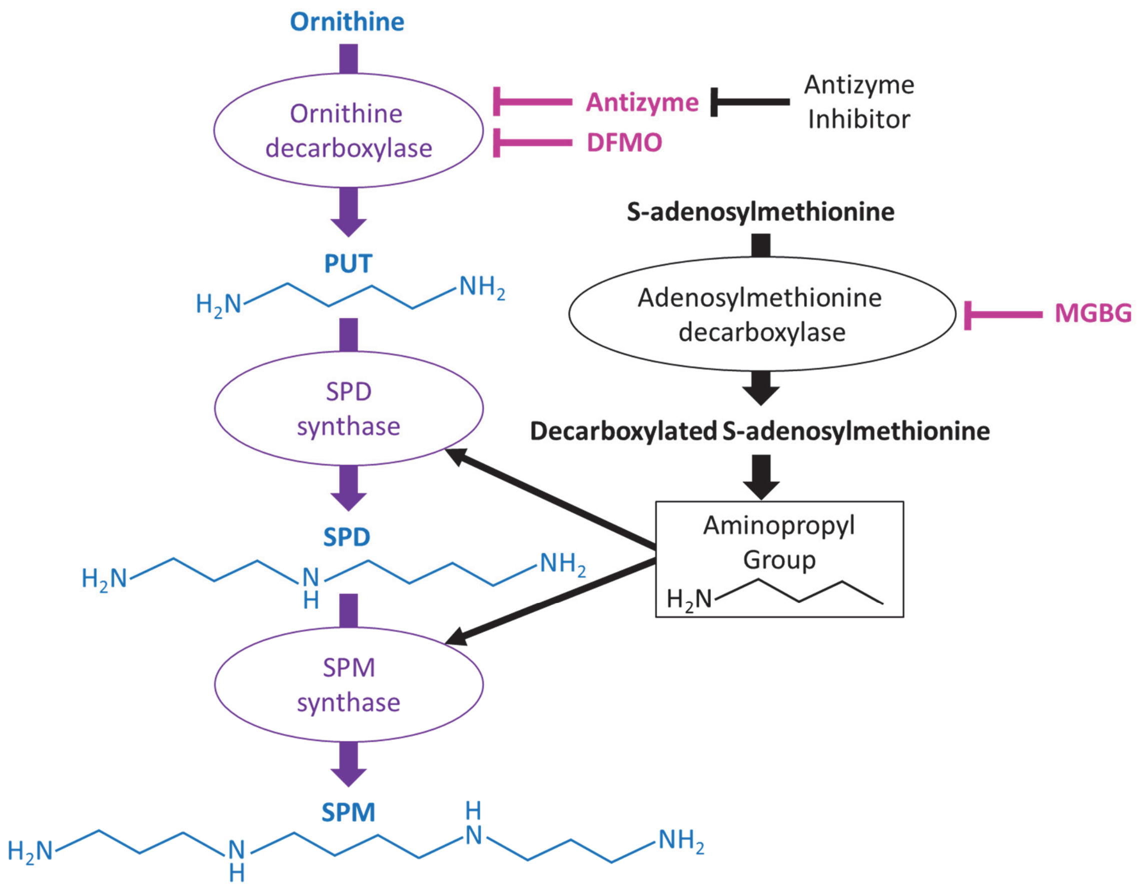

Figure S1. Polyamine biosynthesis pathway and its inhibitors. DFMO, difluoromethylornithine; **MGBG**, methylglyoxal bis-(guanyl hydrazone); **SPM**, spermine; **SPD**, spermidine; **PUT**, putrescine

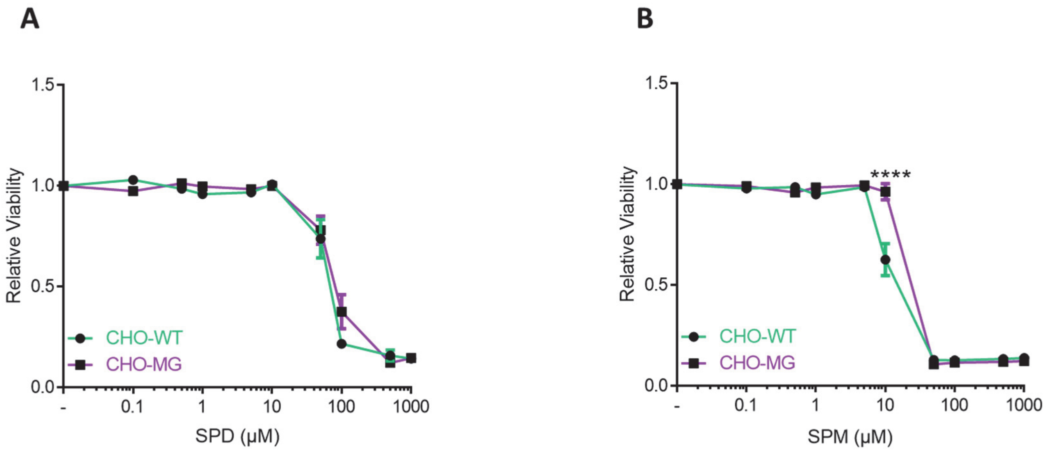

Figure S2. Effect of polyamines on the viability of CHO-MG cells. *A, B,* Cells were treated for 24 h with the indicated concentrations of only SPD *(A)* and SPM *(B)*. CellTiter 96® AQ_{ueous} One Solution Cell Proliferation Assay (MTS) was used to assess cell viability and dose-response curves were plotted (n=3). Data represent mean ± SEM (****P<0.0001) generated with two-way ANOVA and Bonferroni *post hoc* corrections. **SPM**, spermine; **SPD**, spermidine.

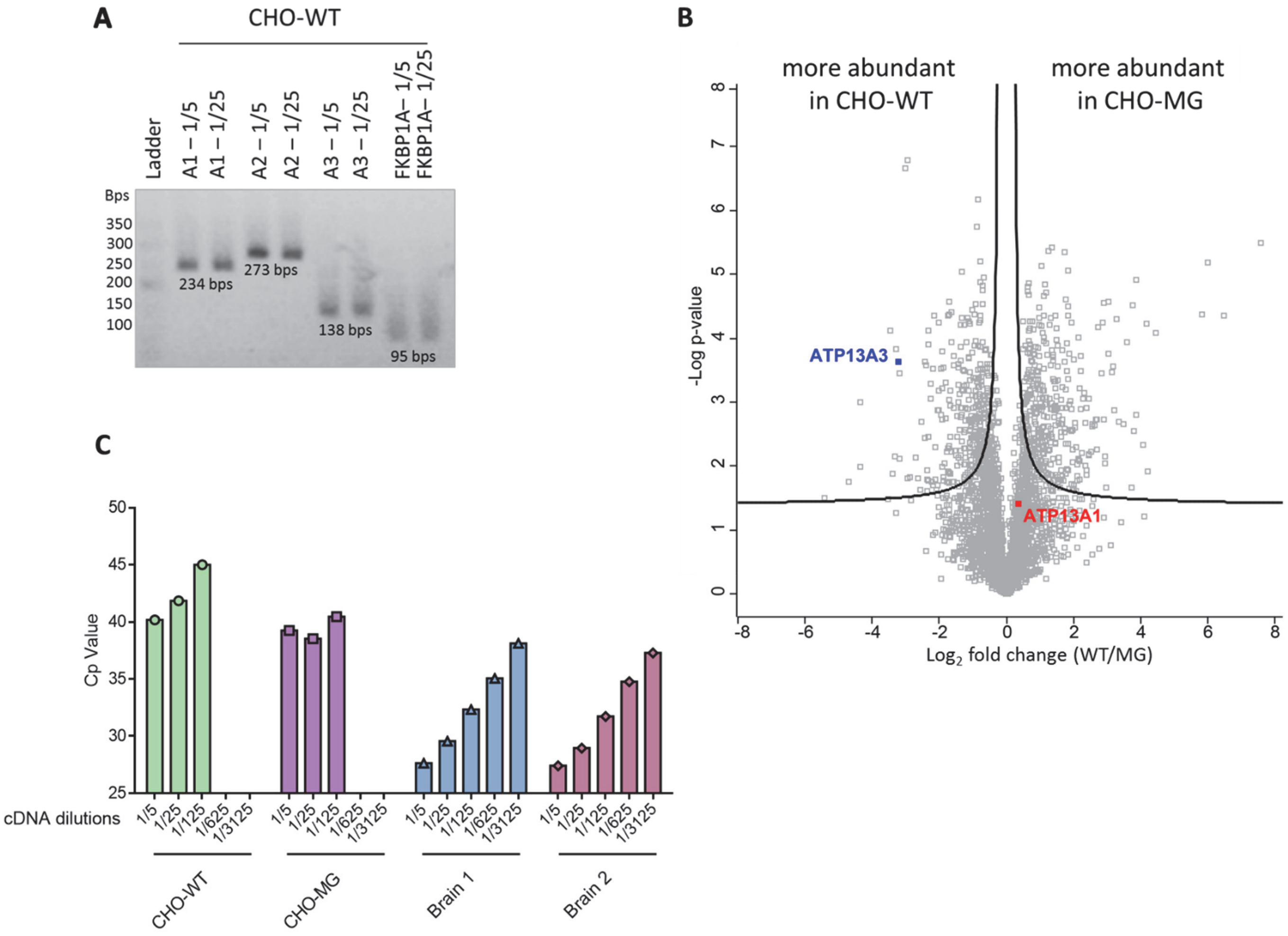

Figure S3. ATP13A3 is downregulated in CHO-MG cells. *A,* ATP13A1-3 mRNA levels were measured with qPCR using SYBR Green master mix and qPCR products from two cDNA dilutions (1/5, 1/25) were run on an agarose gel to check the specificity of the primers. *B,* Proteomic analysis was carried out on membrane fractions of CHO-WT and CHO-MG cells and detected proteins including ATP13A3 were plotted in a volcano plot (n=4). *C,* Cp values, demonstrating the ATP13A4 mRNA primer linearity in hamster brain tissues *versus* the CHO cells, are shown across the indicated cDNA dilutions. **A1-A3**, ATP13A1-3; **FKBPA1**, FKBP prolyl isomerase 1A

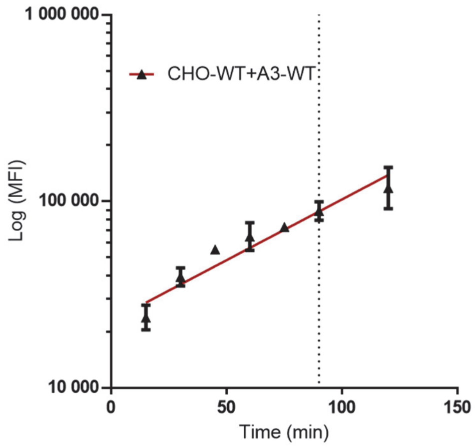

Figure S4. Uptake of BODIPY-PUT uptake falls within the linear phase. Cells were treated with 5 µM BODIPY-PUT at 37°C for the indicated times**.** Uptake was measured in terms of mean fluorescence intensity (MFI) up to 1 x 10^4 events (debris-free) per condition on the flow cytometer. Data represent mean ± SEM. The dashed line refers to the time point that was used for the treatment of BODIPY-polyamines in all flow cytometry experiments (n=2-4). **A3-WT**, overexpression of wild type ATP13A3; **A3-DN**, overexpression of ATP13A3 catalytically dead mutant D498N; **BODIPY**, boron dipyrromethene; **PUT**, putrescine

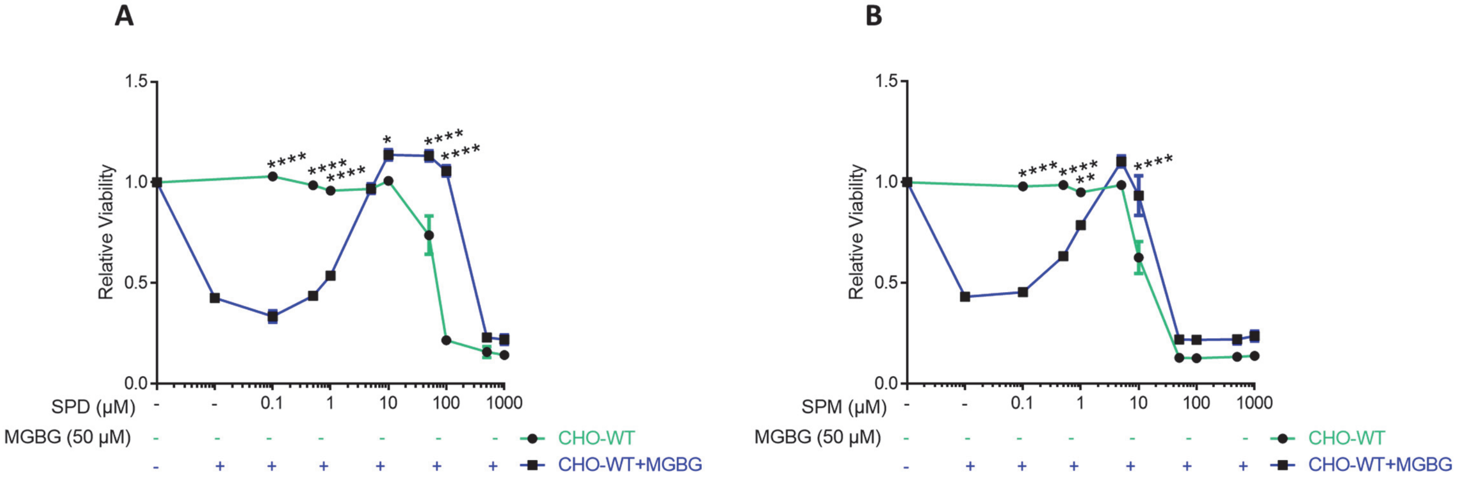

Figure S5. Competition of MGBG with SPD and SPM in CHO-WT cells. *A, B,* Cells were treated for 24 h with the indicated concentrations of SPD (n=3) *(A)* and SPM (n=3) *(B)* with or without 50 µM MGBG co-treatment. CellTiter 96® AQueous One Solution Cell Proliferation Assay (MTS) was used to assess cell viability and dose-response curves were plotted. Data represent mean ± SEM (**P*<0.05, ***P*<0.01, ****P*<0.001, and *****P* <0.0001) generated with two-way ANOVA and Bonferroni *post hoc* corrections. **MGBG**, methylglyoxal bis-(guanyl hydrazone); **SPM**, spermine; **SPD**, spermidine

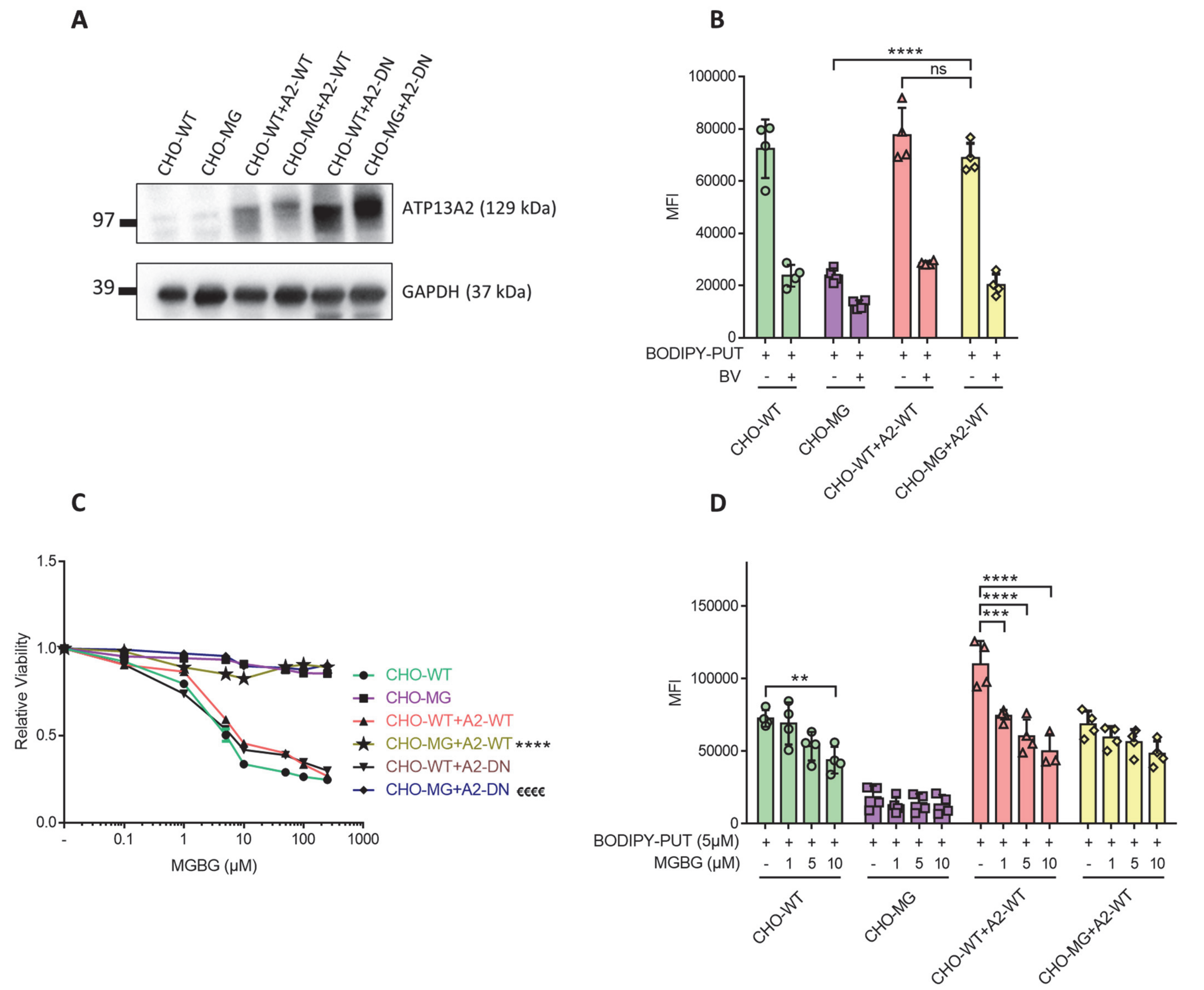

Figure S6. Expression of wild-type ATP13A2 restores polyamine uptake, but it does not affect MGBG resistance in CHO-MG cells. *A,* Stable cell lines were generated by lentiviral transduction to overexpress wild type (A2-WT) or a catalytically dead mutant (A2-DN) of ATP13A2. Expression of the viral vectors was verified by immunoblotting using ATP13A2 selective antibody, while the loading was monitored by house-keeping protein GAPDH selective antibody. *B, D,* Cells were treated with 5 µM BODIPY-PUT alone (B) or combined with MGBG (D) for 90 min at 37°C with or without 90 min of 1 mM BV pre-treatment to inhibit polyamine uptake. Uptake was measured in terms of mean fluorescence intensity (MFI) up to 1 x 10^4 events (debris-free) per condition on the flow cytometer (n=4). *C,* Cells were treated for 24 h with different doses of MGBG. Cell viability was assessed using CellTiter 96® AQ_{ueous} One Solution Cell Proliferation Assay (MTS) and dose-response curves were plotted (n=3). Data represent or mean ± SD *(B, D)* and individual data points (representing replicates) are overlaid on bar graph plots, or mean ± SEM *(C)* (***P*<0.01, $****^{/€€€€}$*P*<0.0001, ns = not significant, **** *vs* CHO-WT+A2-WT, and $^{€€€€}$ *vs* CHO-WT+A2-DN). Analyses were performed using two-way ANOVA and Bonferroni post hoc corrections. **BODIPY**, boron dipyrromethene; **BV**, benzyl viologen; **GAPDH**, Glyceraldehyde 3-phosphate dehydrogenase; **MGBG**, methylglyoxal bis-(guanyl hydrazone); **PUT**, putrescine; **A2-WT**, overexpression of wild type ATP13A2; **A2-DN**, overexpression of ATP13A2 catalytically dead mutant D508N
